# The Identity, Virulence, and Antifungal Effects of the Didymellacesous Fungi Associated with the Rapeseed Blackleg Pathogen *Leptosphaeria biglobosa*

**DOI:** 10.3390/jof9121167

**Published:** 2023-12-04

**Authors:** Junyu Cheng, Tao Luo, Mingde Wu, Long Yang, Weidong Chen, Guoqing Li, Jing Zhang

**Affiliations:** 1 State Key Laboratory of Agricultural Microbiology and Key Laboratory of Plant Pathology of Hubei Province, Huazhong Agricultural University, Wuhan 430070, China; junyucheng@webmail.hzau.edu.cn (J.C.); luotao0503@webmail.hzau.edu.cn (T.L.); mingde@mail.hzau.edu.cn (M.W.); yanglong@mail.hzau.edu.cn (L.Y.); guoqingli@mail.hzau.edu.cn (G.L.); 2 United States Department of Agriculture, Agricultural Research Service, Washington State University, Pullman, WA 99164, USA; weidong.chen@usda.gov

**Keywords:** rapeseed, *Leptosphaeria biglobosa*, *Didymella macrostoma*, penicillither, biological control

## Abstract

Eight fungal strains (P1 to P8) were isolated from rapeseed stems (*Brassica napus*) infected with the blackleg pathogen *Leptosphaeria biglobosa* (Lb). They formed pycnidia with similar morphology to those of Lb, and thus were considered as Lb relatives (LbRs). The species-level identification of these strains was performed. Their virulence on rapeseed and efficacy in the suppression of Lb infection were determined, and the biocontrol potential and biocontrol mechanisms of strain P2 were investigated. The results showed that the LbRs belong to two teleomorphic genera in the family *Didymellaceae*, *Didymella* for P1 to P7 and *Boeremia* for P8. Pathogenicity tests on rapeseed cotyledons and stems indicated the LbRs were weakly virulent compared to *L. biglobosa*. Co-inoculation assays on rapeseed cotyledons demonstrated that P1 to P7 (especially P1 to P4) had a suppressive effect on Lb infection, whereas P8 had a marginal effect on infection by *L. biglobosa*. Moreover, *D. macrostoma* P2 displayed a more aggressive behavior than *L. biglobosa* in the endophytic colonization of healthy rapeseed cotyledons. Cultures of P2 in potato dextrose broth (PDB) and pycnidiospore mucilages exuded from P2 pycnidia showed antifungal activity to *L. biglobosa*. Further leaf assays revealed that antifungal metabolites (AM) of strain P2 from PDB cultures effectively suppressed infection by *L. biglobosa*, *Botrytis cinerea* (gray mold), and *Sclerotinia sclerotiorum* (white mold). An antifungal metabolite, namely penicillither, was purified and identified from PDB cultures and detected in pycnidiospore mucilages of strain P2. This study suggests that the LbRs are a repertoire for screening biocontrol agents (BCAs) against rapeseed diseases, and *D. macrostoma* P2 is a multi-functional BCA, a penicillither producer, and an endophyte.

## 1. Introduction

Rapeseed (*Brassica napus*) is a worldwide crop for edible vegetable oil, honey, livestock feedstuff, and green manure. In China, rapeseed has been widely cultivated both in the south as a winter crop and in the north as a spring crop, and has become the most important oil crop. The annual planting acreage has reached ~7 × 10^6^ ha, and the annual seed yield has reached ~1.3 × 10^7^ metric tons [1].

Like other crops, rapeseed encounters many fungal pathogens during growth and development, which cause diseases, resulting in substantial seed yield losses. Several rapeseed pathogens, such as *Botrytis cinerea* (gray mold), *Leptosphaeria biglobosa* (blackleg), *L. maculans* (blackleg), and *Sclerotinia sclerotiorum* (stem rot), are the top biotic threats worldwide to the rapeseed industry [2]. The control of blackleg (*L. maculans*) largely depends on the use of resistant varieties [3]. However, rapeseed cultivars usually lose ‘resistance’ to this disease after a certain period of cultivation due to the emergence of new races in the pathogen populations [4]. Furthermore, the *Rlm* gene in rape is not resistant to *L. biglobosa*, and no avirulent gene has been found in *L. biglobosa* [5]. On the other hand, the control of gray mold and Sclerotinia stem rot largely depends on the use of fungicides, as highly resistant rapeseed cultivars to *B. cinerea* and *S. sclerotiorum* are not yet available [6,7]. However, although fungicides are effective in most cases, the emergence of fungicide resistance renders fungicides ineffective for the control of these two diseases. Additionally, increasing public concerns over environmental pollution and fungicide residues in agricultural products warrant environmentally friendly alternative approaches to control these diseases. Therefore, it is necessary to develop new control measures (e.g., biological control) against rapeseed diseases.

Biological control using indigenous bacterial and fungal strains has been considered as a safe and sustainable approach for controlling plant diseases. Many biocontrol agents (BCAs) against rapeseed diseases have been reported. Mycoparasites such as *Coniothrium minitans* and *Trichoderma* spp. are effective BCAs against Sclerotinia stem rot [8,9]. Bird’s nest fungi such as *Cyathus striatus,* as well as bacterial antagonists such as *Bacillus amyloliquefaciens*, *Pseudomonas chlororaphis,* and *Serratia plymuthica,* have promising potential to control blackleg in rapeseed [10,11,12]. A few strains of *Bacillus* spp., *C. minitans,* and *Trichoderma* spp. have been successfully developed as commercial biofungicides [8,9]. They were mainly used for the control of diseases of high-value crops, such as vegetables, but rarely used for the control of rapeseed diseases, possibly due to low and inconsistent control efficacy, the narrow control spectrum, and the high cost. Therefore, it is necessary to explore novel BCAs to overcome these shortages.

Fungi in the genus *Phoma* and their related teleomorphs such as *Didymella* are widely distributed in diverse ecological niches associated with plants as plant endophytes, saprophytes, or pathogens [13,14,15]. More importantly, *Phoma* species are important sources of antimicrobial metabolites, which can be potentially exploited for the control of plant diseases [16]. For example, *Phoma glomerata* (telemorph: *Didymella glomerata*) was found to be an effective BCA against clubroot in cruciferous crops caused by *Plasmodiophora brassicae* through the production of cyclic tetramic acids [17,18,19]. Meanwhile, Sullivan et al. (2000) reported that the *Phoma glomerata* can also parasitize powdery mildew, which can produce a large number of pycnidia on cleistothecia, thus inhibiting the growth of powdery mildew [20]. Furthermore, the antifungal substances produced by the endophytic *Phoma* can inhibit the growth of *Sclerotinia sclerotiorum*, as well as *Verticillium dahliae*, the causal agent of verticillium wilt in malvaceous crops [21,22].

In our recent survey of the pathogens for blackleg in rapeseed in China, stems of rapeseed showing typical blackleg symptoms were collected from various rapeseed-producing areas in China [23]. A total of 6015 fungal strains were obtained; the majority of the strains (6007 strains, accounting for 99.9%) were *L. biglobosa* [23], and the remaining eight strains (P1 to P8) were considered to be *L. biglobosa* relatives (LbR) as they shared the same ecological niche (e.g., rapeseed stems) with *L. biglobosa* and formed black pycnidia and pycnidiospores that are morphologically similar to those formed by *L. biglobosa*. The peculiar association of the LbR strains with the blackleg disease promoted us to pose a few questions: Are the LbR strains as virulent as *L. biglobosa*/*L. maculans* against rapeseed? If they are not pathogens of rapeseed, do they have any biocontrol potential? What role can the LbR strains play in the rapeseed disease system with pathogens and plants? To address these questions, we conducted this study and reported herein results about the taxonomic identity of the eight LbR strains and some important biological characteristics, such as virulence on rapeseed and interaction with *L. biglobosa* and rapeseed. The results suggest that the LbR strains are an important source for screening BCAs against *L. biglobosa* and other rapeseed pathogens, and *D. macrostoma* P2 is a multifunctional BCA, a producer of antifungal metabolites, and an endophyte of rapeseed.

## 2. Materials and Methods

### 2.1. Fungal Strains and Cultural Media

A total of 25 fungal strains were used in this study, including 8 LbR strains (P1 to P8) and 15 *Leptosphaeria* strains (Table 1) as well as *B. cinerea* B05.10 and *S. sclerotiorum* Ss-1. The origins of the *Leptosphaeria* strains and the LbR strains are indicated in Table 1. *B. cinerea* B05.10 was provided by Dr. Zhonghua Ma of Zhejiang University (Hangzhou, China), and *S. sclerotiorum* Ss-1 was isolated from rapeseed. Five cultural media, including malt extract agar (MEA), oatmeal agar (OA), potato dextrose agar (PDA), potato dextrose broth (PDB), and water agar (WA), were used in this study. They were prepared using the procedures described by Fang (1998).

### 2.2. DNA Extraction, PCR, and Sequencing

The 8 LbR strains and the 15 *Leptosphaeria* strains were incubated on PDA overlaid with cellophane membrane; the cultures were incubated at 20 °C in the dark for 4 d. Mycelia of each strain were harvested using a sterilized aluminum spatula. Genomic DNA (gDNA) was extracted from the mycelia of each strain using the CTAB method and dissolved in Tris-EDTA (TE) buffer. DNA solutions (~50 ng/mL) were stored at −80 °C until use.

The gDNA was used as a template for the PCR amplification of the DNA sequences of ITS (internal transcribed spacer, ITS1-5.8S-ITS2), LSU (large subunit of 28S rRNA), *tub2* (tublin), and *rpb2* (the second subunit of RNA polymerase) using the primer pairs V9G/ITS4, LR0R/LR7, TUB2Fd/TUB4Rd, and fRPB2-5F/fRPB2-7cR, respectively (Table S1). The PCR mixture (25 μL) contained: 2.5 μL of 10× EasyTaq buffer (TransGen Biotechnol. Co., Ltd., Beijing, China), 50 μmol/L of dNTPs, 0.1 μmol/L of each primer, 0.75 U of Taq DNA polymerase (TaKaRa Biotechnol. Co., Ltd., Dalian, China), 50 ng of DNA template, and ddH_2_O. The PCR was performed in a S1000^TM^ Thermal Cycler (Bio-Rad Laboratories, Hercules, CA, USA) with the thermal programs listed in Table S2. The PCR products were cloned and sequenced using routine procedures [24]. The DNA sequences were deposited in the GenBank public database (http://www.ncbi.nlm.nih.gov (accessed on 28 November 2023)), and their GenBank accession numbers are listed in Table 1.

### 2.3. Phylogenetic Analysis

Multiple DNA sequences of ITS, LSU, *tub2,* and *rpb2* for 36 fungal taxa (Table 1 and Table S3) were aligned using Clustal W in MEGA X [25] with default settings. The dataset was used to construct phylogenetic trees using the procedures described by Guo et al. [26]. Bayesian inference (BI) was used to construct phylogenies using MrBayes v. 3.1.2 [27]. The best-fit model for nucleotide substitution in each partition was determined as GTR + I + G using MrModeltest v. 2.3 [28]. Two analyses of four Markov Chain Monte Carlo (MCMC) chains were conducted from random trees with 6 × 10^6^ generations. The analyses were sampled every 1000 generations, which were stopped once the average standard deviation of split frequencies was below 0.01. The first 25% of the trees were discarded as the burn-in phase of each analysis, and the remaining trees were summarized to calculate the posterior probabilities (PPs) of each clade being monophyletic.

Additionally, maximum parsimony analyses (MPs) were performed on the multi-locus alignment using PAUP (Phylogenetic Analysis Using Parsimony) v. 4.0b10 [29]. Phylogenetic trees were generated using the heuristic search option with Tree Bisection Reconnection (TBR) branch swapping and 1000 random sequence additions. Maximum trees were set up to 5000, branches of zero length collapsed, and all multiple parsimonious trees were saved. Clade stability was assessed using a bootstrap analysis with 1000 replicates. Then, tree length (TL), consistency index (CI), retention index (RI), rescaled consistency index (RC), and homoplasy index (HI) were calculated. Furthermore, raxmlGUI-2.0.0 [30] was used for maximum likelihood (ML) analysis. The analysis was performed with a GTR site substitution model. The branch support was evaluated with a bootstrapping (BS) method of 1000 replicates [31]. The phylogenetic tree was visualized in FigTree v. 1.4.2 [32] and deposited in TreeBASE (https://treebase.org/treebase-web/home.html (accessed on 28 November 2023)) with the accession number Study 27919.

In order to further define subspecies status of the 15 *Leptosphaeria* strains, another dataset containing DNA sequences of ITS and LSU was established to accommodate 38 fungal taxa (Table 1 and Table S3). It was used to construct another phylogenetic tree with the same procedures described above. The tree was also deposited in TreeBASE with the accession number Study 27916.

### 2.4. Morphological Characterization

The eight LbR strains (P1 to P8) and *L. biglobosa* Lb20 were cultured on MEA, OA, and PDA in Petri dishes (90 mm in diameter; 20 mL of medium in each dish), with five dishes (replicates) for each strain. The cultures were incubated at 20 °C in the dark, the diameter of each fungal colony was measured after incubation for 4 and 6 d, and the data were used to calculate the radial mycelial growth rate, expressed as mm per day. After incubation for 7 to 14 d, the morphological features of the colonies were observed based on description by Chen et al. [33]. The size of randomly selected 20 pycnidia and 50 pycnidiospores for each strain was measured under a compound light microscope (Model Nikon 80i, Tokyo, Japan). To observe the morphology of pycnidia, a mycelial agar plug with mature pycnidia was carefully removed from a 14-day-old PDA culture; it was then immersed in formalin–acetic acid–alcohol (FAA) fixative solution (formalin/glacial acetic acid/50% ethanol, 5/5/9, *v*/*v*/*v*). The specimens were dehydrated with serial ethanol solutions (70% to 100%, *v*/*v*), embedded in paraffin wax, and sectioned. The resulting thin slices were mounted on glass slides, followed by de-waxing, rehydration, hematoxylin–eosin staining, and observation under the compound light microscope.

### 2.5. Virulence Determination

The virulence of the eight LbR strains was determined on cotyledons and basal stems of rapeseed (*B. napus* cultivar Zhongshuang No.9) in comparison with *L. biglobosa* Lb20. The nine strains were incubated on PDA at 20 °C for 15 d; pycnidisopores were harvested by washing the PDA cultures with sterile distilled water for the preparation of pycnidiospore suspensions (1 × 10^7^ spores/mL), which were used as inoculum in the following two trials.

In the cotyledon inoculation trial, rapeseed seeds were pre-germinated at 20 °C on moisturized filter papers for 3 d. The germinated seeds were then sown in plant potting mix (N:P:K = 1:1:1, pH 6) in plastic pots (10 × 10 cm, diameter × height), and the pots were maintained for 14 d in a growth room (20 °C, 16 h light/8 h dark) and watered as needed. The cotyledons were wounded using a needle, with two wounds (one on each side of the main vein) per cotyledon. An aliquot (10 μL) of the pycnidiospore suspension of each strain was applied on the wounds and surrounding area, with 16 wounds on eight cotyledons (4 seedlings) for each strain. For the control treatment, sterile water (10 μL) was applied to 16 wounds on eight cotyledons. The treated seedlings were transferred to a plastic container in a growth room (20 °C, 16 h light/8 h dark) to maintain high humidity (~100% R.H.). The lesion diameter around each wound was measured at 7 d post-inoculation (dpi).

In the stem inoculation trial, the pycnidiospore suspensions of each strain and water alone (control) were inoculated into pre-wounded basal stems of 60-day-old rapeseed plants using a micropipette, with 10 μL on each wound and six stems for each strain and control. Then, the basal stems were wrapped at the wound areas with plastic film, and the treated plants were maintained outside (15 °C to 25 °C) for 21 d; the lesion length and width on the stem epidermis and inside the stem pith were measured around each wound.

### 2.6. Co-Inoculation Assays

Assays were carried out to determine the effects of the LbR strains on infection by *L. biglobosa*. The pycnidiospore suspensions (1 × 10^7^ spores/mL) of the LbR strains and *L. biglobosa* Lb20 were prepared as mentioned above. There were six treatments for each combination of an LbR strain and Lb20, namely six different ratios of pycnidiospore suspensions of an LbR strain and Lb20 at 2:8, 3:7, 5:5, 7:3, 8:2, and 10:0 (LbR:Lb20, *v*/*v*). Meanwhile, six control treatments, namely six mixtures of sterile water and pycnidiospore suspension of Lb20 with the ratios at 2:8, 3:7, 5:5, 7:3, 8:2, and 10:0 (water:Lb20, *v*/*v*), were separately set up. The mixture (10 μL) for each treatment was inoculated on 12 wounded cotyledons on six rapeseed seedlings (14 days old). The treated seedlings were maintained in the growth room (20 °C, 100% R.H.) for 7 d, and the lesion diameter around each wound was measured. The assay was repeated three times.

An additional experiment was carried out to assess the association of endophytic growth of P2 with its suppressive effect on infection by *L. biglobosa* Lb20. The mixture of pycnidiospore suspensions (1 × 10^7^ spores/mL) of P2 and Lb20 (1:1, *v*/*v*), as well as the mixture of water and the pycnidiospore suspension of Lb20 (1:1, *v*/*v*), was separately set up and inoculated at wounded cotyledons, with 10 μL on each wound and 80 wounds on 20 seedlings for each treatment. The treated seedlings were incubated in the growth chamber (20 °C) for 7 d, and the lesion diameter around each wound was measured. After measuring the lesion size, the endophytic growth of P2 and Lb20 in the cotyledons in the treatment of P2 and Lb20 in all of the three trials and in the cotyledons in the treatment of water and Lb20 in the third trial was zoned around the center of each wound towards the margin: 10 mm in width for each zone and three zones (Z1, Z2, Z3) on each cotyledon. The tissue of each zone was cut into small pieces (3 × 3 mm in size) using a sterilized razor blade; the resulting cotyledon pieces (CP) were surface-disinfected with 5% NaClO and placed on acidified PDA, with 8 CPs in each dish and 40 CPs in total for each zone. The dishes were placed in an incubator (20 °C) for 5 d; fungal colonies formed around the CPs were identified based on the colony morphology of P2 and Lb20. The isolation frequency of each fungus was calculated based on the number of CPs with that fungus and the total number of CPs used in isolation. The experiment was repeated three times as three trials in this assay.

### 2.7. Suppression of Fungal Infection by Cultural Filtrates of LbR Strains

The LbR strains were shake-cultured (20 °C, 150 rpm) in PDB for 15 d. The cultural filtrate (CF) of each strain was collected by filtering the cultures through four-layer cheesecloth, followed by centrifuging at 6000 rpm for 5 min to remove hyphal fragments. The resulting supernatant was used to treat rapeseed leaves of 30-day-old plants. Three leaves on a plant were selected and wounded with a needle (two wounds on each leaf (one on each side of the main vein)). Filter paper disks (5 mm in diameter) were placed on the wounds to help hold the CF as well as the pycnidiospore suspension of *L. biglobosa* Lb20. The CF of each strain or fresh PDB (control) was applied to the filter paper disks, with 20 μL of CF on each disk and 15 leaves for each strain and control. Three hours later, aliquots (10 μL) of the pycnidiospore suspension (1 × 10^7^ spores/mL) of *L. biglobosa* Lb20 were pipetted onto the filter paper disks that were pre-treated with the CF or water. The inoculated seedlings were maintained under humid conditions (100% R.H.) in the growth room (20 °C) for 7 d. The leaf lesion diameter around each filter paper disk was measured. The bioassay was repeated three times.

The P2 CF was further tested for the suppression of infection by *S. sclerotiorum* Ss-1 and *B. cinerea* B05.10 on rapeseed leaves. There were four treatments: P2 CF+Ss-1, Ss-1 alone (Control 1), P2 CF+B05.10, and B05.10 alone (Control 2). In the treatments of P2 CF+Ss-1 and P2 CF+B05.10, the P2 CF was sprayed evenly on leaves of 30-day-old rapeseed plants, with ~0.5 mL of P2 CF or PDB on each leaf. In the treatments of Ss-1 alone and B05.10 alone (Control 1 and Control 2), fresh PDB (~0.5 mL per leaf) was sprayed evenly on leaves of rapeseed. There were 12 leaves on four plants for each treatment. Three hours later, the leaves were inoculated with mycelial agar plugs (5 mm in diameter) of *S. sclerotiorum* Ss-1 or *B. cinerea* B05.10, with two mycelial agar plugs (one on each side of the main vein) per leaf. The inoculated plants were maintained in a moist chamber (20 °C, 16 h light/8 h dark) for 3 d, and the leaf lesion diameter around each mycelial agar plug was measured. The bioassay was repeated two times.

### 2.8. Determination of the Antifungal Activity of P2 Cultural Filtrate

Two methods, agar diffusion and agar amendment, were used to determine the antifungal activity of P2 CF from 15-day-old PDB cultures. The agar diffusion method was used to determine the suppression of pycnidiospore germination of *L. biglobosa* Lb20. The pycnidiospore suspension (1 × 10^8^ spores/mL) of *L. biglobosa* was incorporated into PDA at the ratio of 1:9 (*v*/*v*), and the spore-containing medium was distributed in Petri dishes (~20 mL in a 9 cm diameter dish). Sterilized Oxford cups (10 × 6 mm, height × inner diameter) were placed on the spore-containing PDA in the dishes, with two cups in each dish and three dishes as six replicates (cups) for each treatment. The P2 CF or fresh PDB (control) was pipetted into the Oxford cups (200 µL per cup). The dishes were maintained in an incubator (20 °C) for 4 d, and the diameter of the inhibition zone around each cup was measured.

The agar amendment method was used to determine the suppression of mycelial growth of *L. biglobosa*, *S. sclerotiorum,* and *B. cinerea*. The P2 CF from the 15-day-old PDB cultures was incorporated into PDA at concentrations of 0% (Control), 0.1%, 0.6%, 1.3%, and 5.0% (*v*/*v*), and PDA alone was treated as control. The mycelial agar plugs of each target fungus were inoculated on PDA supplemented with P2 CF or on PDA alone (control), with one mycelial agar plug per dish and three dishes (replicates) for each treatment. The cultures were incubated at 20 °C, and fungal colony diameters were measured at 3 d for *B. cinerea* and *S. sclerotiorum* and at 15 d for *L. biglobosa*, and the diameter of the fungal colony in each dish was measured. The percentage of colony size reduction (CSR) to indicate mycelial growth inhibition rate by P2 CF was calculated using the following formula:

CSR (%) = 100% × (AD_CK_ − D_CF_)/AD_CK_

where AD_CK_ represents the average colony diameter of a target fungus in the control treatment, and D_CF_ represents the colony diameter of that fungus in the treatment of P2 CF at a given concentration.

### 2.9. Determination of the Antifungal Activity of P2 Pycnidiospore Suspensions

Strain P2 was inoculated on PDA at 20 °C in the dark for 15 d. Sterile distilled water was added to the cultures, and pycnidiospores in mucilages exuding from pycnidia were washed off by gently scraping the cultures using a sterilized glass rod. The spore mixture was filtered through four-layer cheesecloth to remove hyphal fragments and obtain pycnidiospore suspension. The concentration of pycnidiospores was measured using a hemocytometer and adjusted to 1 × 10^8^ spores/mL with sterile distilled water. The pycnidiospore suspension was centrifuged at 6000 rpm for 10 min to precipitate the pycnidiospores. The resulting supernatant contained substances from pycnidiospore mucilages. It was designated here as P2 PS (e.g., pycnidiospore-associated substances). The antifungal activity of P2 PS towards *L. biglobosa* Lb20 and *D. macrostoma* P2 itself was then tested using the agar diffusion method mentioned above with water as the control.

### 2.10. The Extraction and Purification of Antifungal Metabolites

The antifungal metabolites in P2 CF were extracted using the chloroform isolation method outlined in Figure S1. A brown powder (~8 g) of the chloroform extract (P2 CE) was obtained. It was dissolved in 10 mL of methanol, and the resulting solution was subjected to silica gel chromatography using a mixture of chloroform and methanol as gradient eluents. A total of 20 serial fractions (200 mL for each) were obtained and vacuum-lyophilized. Then, the dried fractions were separately dissolved in methanol, and the antifungal activity of the resulting solutions was tested using the agar diffusion method mentioned above with *L. biglobosa* as a bio-indicator.

### 2.11. Identification of Penicillither

The fraction Fr.4 (Figure S1) was further purified by semi-preparative HPLC, and a pure sub-fraction Fr.4.4 was obtained (Figure S1). Electrospray ionization mass spectrometry (ESI-MS), ultraviolet absorption, and nuclear magnetic resonance spectroscopy (NMR) were used to determine the chemical formula and structure of Fr.4.4. The ESI-MS analysis was performed on Waters ACQUITY UPLC coupled with ACQUITY UPLC^®^ BEH C18 column (1.7 μm, 2.1 mm × 50 mm). Fr.4.4 was dissolved in methanol, and the resulting solution (1 μg/mL, 1 μL) was injected into the HPLC instrument. The column was eluted with mixtures of flow phases A (100% acetonitrile) and B (99.9% water + 0.1% formic acid, *v*/*v*). The operating parameters were set as foolows: capillary voltage at 2.0 kV, cone voltage at 30 kV, Z-spray source temperature at 120 °C, desolvation temperature at 450 °C, gas flow at 800 L/h, mass range from 50 to 1200 *m/z*. The mass spectrum was collected in negative and positive modes and used to determine the element composition of Fr.4.4 using Waters mass spectrometry software Masslynx v4.1. The resulting information about molecular mass and formula was searched in CAS SciFinder^®^ (https://scifinder.cas.org (accessed on 28 November 2023)) to confirm chemical identity.

To determine the ultraviolet absorption spectrum, the methanol solution of Fr.4.4 (100 μg/mL) was pipetted into a colorimetric tube (500 μL), which was placed in a UV2600 UV-VIS photometer (Shimadzu, Kyoto, Japan). The ultraviolet absorption spectrum of the solution at 200 to 700 nm was then determined.

To determine NMR spectra, Fr.4.4 was dissolved in 500 μL of DMSO-d6 (Sigma-Aldrich^®^, St. Louis, MO, USA), and the resulting solution (6 μg/μL) was pipetted into a nuclear magnetic tube (5.0 × 177.8 mm, Sigma-Aldrich^®^), which was then placed in a full digital superconducting NMR spectrometer (AVANCE III 600 MHz, Brucker, Karlsruhe, Germany) to determine ^1^H-NMR and ^13^C-NMR spectra. Tetramethylsilane (0.03%, *w*/*v*) was used as the internal standard in NMR.

### 2.12. The Quantification of Penicillither

Solutions of purified penicillither at five concentrations (6.2, 12.5, 25.0, 50.0, 100.0 μg/mL) were prepared. They were separately injected (1 μL for each solution) in Shimadzu LC-20AT HPLC with a Thermo Scientific UMISil C18 (Thermo Scientific, Waltham, MA, USA) column (5 μm, 250 × 4.6 mm) and Shimadzu SPD-20A UV detector. The compounds were eluted using the same elution phases and programs mentioned above to collect the absorbance peaks; the data, together with concentrations of the two compounds, were used to establish standard curves. Then, P2 CF, P2 CE, and P2 PS (1.0 × 10^8^ spores/mL) were separately injected into HPLC to determine the absorbance peaks of penicillither, and the data were used to calculate the content or concentration of these two compounds with the standard curves as a reference.

### 2.13. Data Analysis

Data for different treatments in each experiment or bioassay were analyzed using the procedure of Analysis of Variance (ANOVA) in the SAS software (SAS Institute, Cary, NC, USA, V. 8.0, 1999). The percentage data on colony size reduction (CSR) and lesion size reduction (LSR) by the cultural filtrates (CFs) of the LbR strains were transformed by multiplying the original data by 100 before ANOVA. After ANOVA, they were back-transformed to percentage values. The treatment means of each parameter in different treatments were separated using the least significant difference (LSD) test (*α* = 0.05). Data on leaf lesion diameters caused by *B. cinerea* (or *S. sclerotorum*) in the treatments of P2 CF and control were analyzed using the procedure Univariate in SAS. The average leaf lesion diameters between P2 CF and control were compared using Student’s *t* test at *α* = 0.05 or 0.01.

## 3. Results

### 3.1. The Species Identity of the LbRs

**Figure 1:**
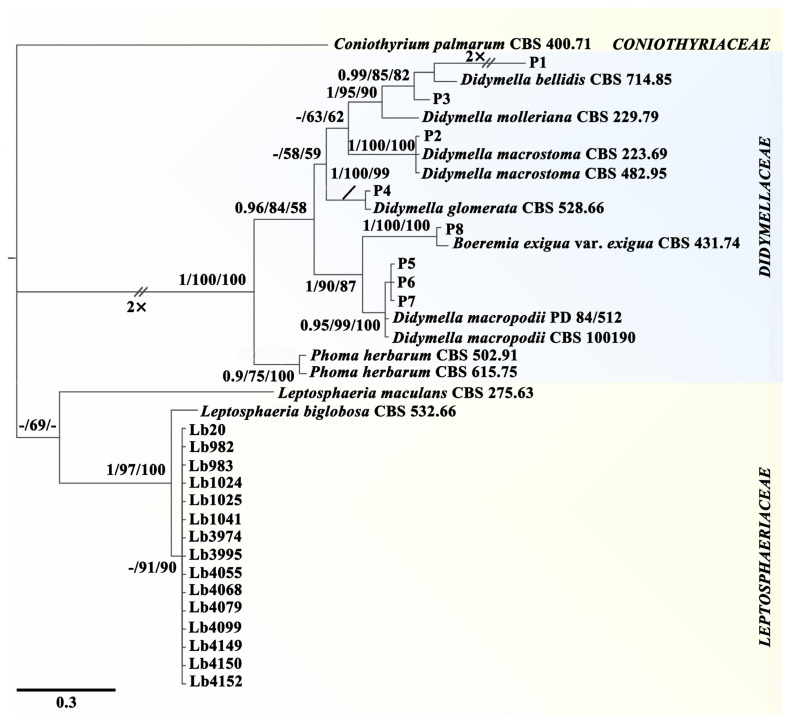
A Bayesian inference phylogenetic tree of 36 fungal taxa. The tree was constructed using concatenated sequences of ITS, LSU, *rpb2,* and *tub2*. Bayesian posterior probability (PP ≥ 0.90), RAxML bootstrap support values (ML ≥ 50%), and MP bootstrap support values (MP ≥ 50%) are shown at the nodes (PP/ML/MP). Scale bar indicates 0.3% sequence divergence.

Two phylogenetic trees were constructed based on the combined sequences of ITS, LSU, *tub2,* and *rpb2* for 36 fungal taxa as well as the combined sequences of ITS and LSU for 38 fungal taxa. Both trees appeared very similar in topology. They consisted of two clades corresponding to two families, *Didymellaceae* and *Leptosphaeriaceae* (Figure 1 and Figure S2). The 8 LbR strains as well as the 10 reference strains of *Boeremia exigua*, *Didymella* spp., and *Phoma herbarum* were located in the clade of *Didymellaceae.* Strains P1 and P3 are closely related to *D. bellidis*, and they formed two unique branches distinct from that for *D. bellidis*, suggesting that they might be two novel species of *Didymella*; strains P2 and P4 are closely related to *D. macrostoma* (anamorph: *Phoma macrostoma*) and *D. glomerata* (anamorph: *Phoma glomerata*), respectively; strains P5, P6, and P7 are closely related to *D. macropodii* (anamorph: *Phoma nigrificans*); and strain P8 is closely related to *Boeremia exigua* (anamorph: *Phoma exigua*). The phylogenetic analysis also showed that 15 strains of *Leptosphaeria* were located in the clade of *Leptosphaeriaceae* (Figure 1 and Figure S2). They are closely related to the sub-clade of *L. biglobosa* ‘brassicae’.

### 3.2. Morphological Characteristics

**Figure 2:**
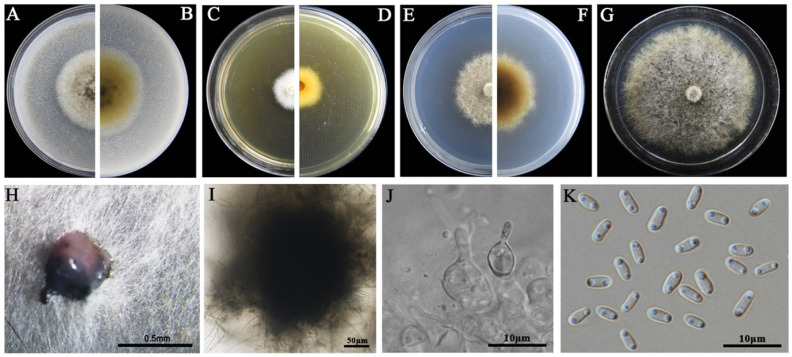
Morphology of *L. biglobosa* Lb20. (**A**,**B**) Colony on OA (front and reverse); (**C**,**D**) colony on MEA (front and reverse); (**E**,**F**) colony on PDA (front and reverse). The cultures in (**A**–**F**) were incubated at 20 °C for 7 days; (**G**) colony on PDA (20 °C, 14 d); (**H**) pycnidia formed on PDA (note the pink pycnidiospore ooze on the surface of the pycnidium); (**I**) a pycnidium formed on PDA; (**J**) conidiogenous cells; (**K**) pycnidiospores (conidia).

**Figure 3:**
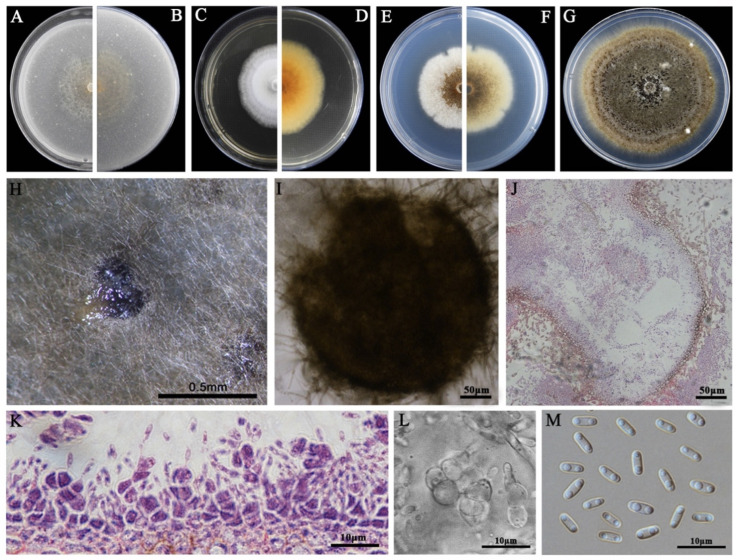
Morphology of *Didymella* sp. P1. (**A**,**B**) Colony on OA (front and reverse); (**C**,**D**) colony on MEA (front and reverse); (**E**,**F**) colony on PDA (front and reverse). The cultures in (**A**–**F**) were incubated at 20 °C for 7 days; (**G**) colony on PDA (20 °C, 14 d); (**H**) pycnidia formed on PDA (note the light yellow pycnidiospore ooze on the surface of the pycnidium); (**I**) a pycnidium formed on PDA; (**J**,**K**) sections of pycnidia; (**L**) conidiogenous cells; (**M**) pycnidiospores (conidia).

**Figure 4:**
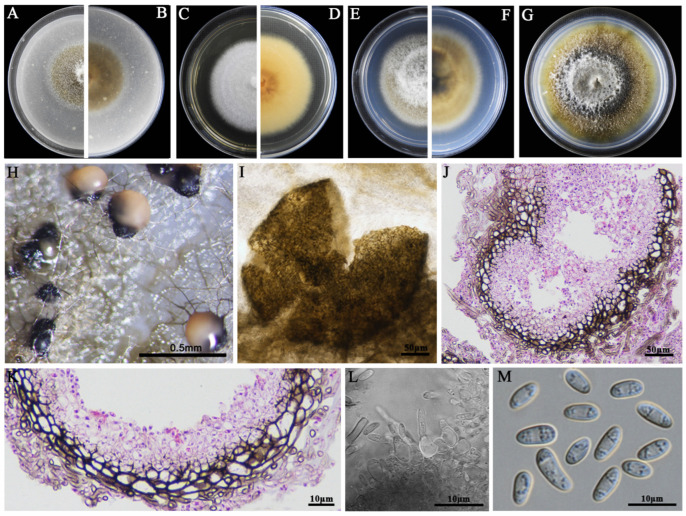
Morphology of *D. macrostoma* P2. (**A**,**B**) Colony on OA (front and reverse); (**C**,**D**) colony on MEA (front and reverse); (**E**,**F**) colony on PDA (front and reverse). The cultures in (**A**–**F**) were incubated at 20 °C for 7 d; (**G**) colony on PDA (20 °C, 14 d); (**H**) pycnidia formed on PDA; (**I**) a crushed pycnidium formed on PDA; (**J**,**K**) sections of two pycnidia; (**L**) conidiogenous cells; (**M**) pycnidiospores (conidia).

**Figure 5:**
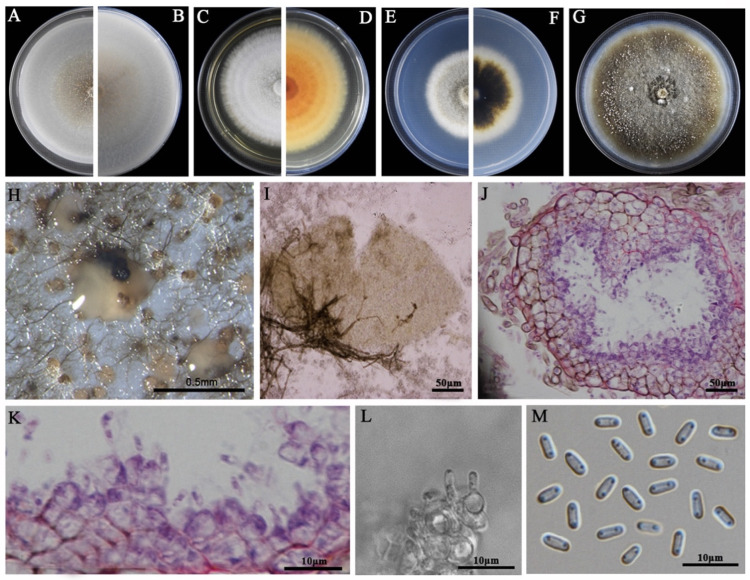
Morphology of *Didymella* sp. P3. (**A**,**B**) Colony on OA (front and reverse); (**C**,**D**) colony on MEA (front and reverse); (**E**,**F**) colony on PDA (front and reverse). The cultures in (**A**–**F**) were incubated at 20 °C for 7 days; (**G**) colony on PDA (20 °C, 14 d); (**H**) pycnidia formed on PDA (note the purulent pycnidiospore ooze on the surface of the pycnidium); (**I**) a crushed pycnidium; (**J**,**K**) sections of pycnidia; (**L**) conidiogenous cells; (**M**) pycnidiospores (conidia).

**Figure 6:**
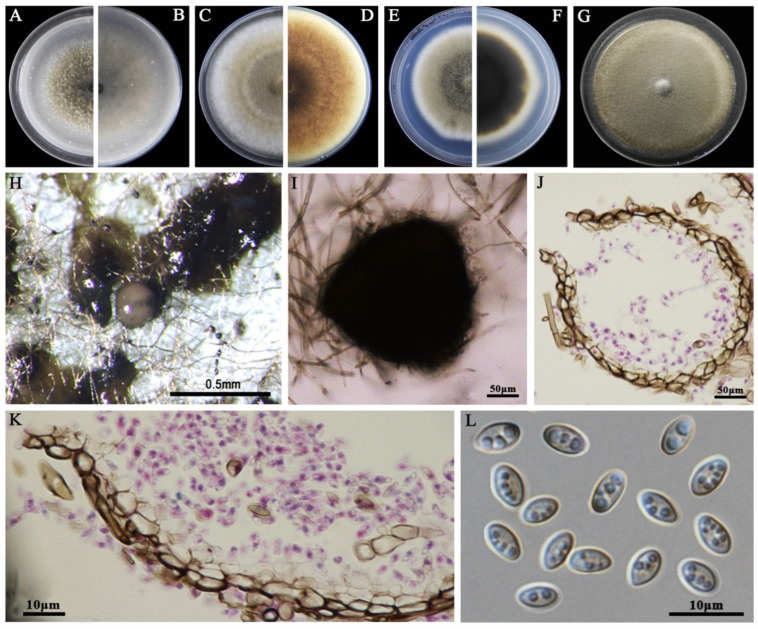
Morphology of *D. glomerata* P4. (**A**,**B**) Colony on OA (front and reverse); (**C**,**D**) colony on MEA (front and reverse); (**E**,**F**) colony on PDA (front and reverse). The cultures in (**A**–**F**) were incubated at 20 °C for 7 days; (**G**) colony on PDA (20 °C, 14 d); (**H**) pycnidia formed on PDA (note the light purple pycnidiospore ooze on the surface of the pycnidium); (**I**) a pycnidium formed on PDA; (**J**,**K**) sections of pycnidia; (**L**) pycnidiospores (conidia).

**Figure 7:**
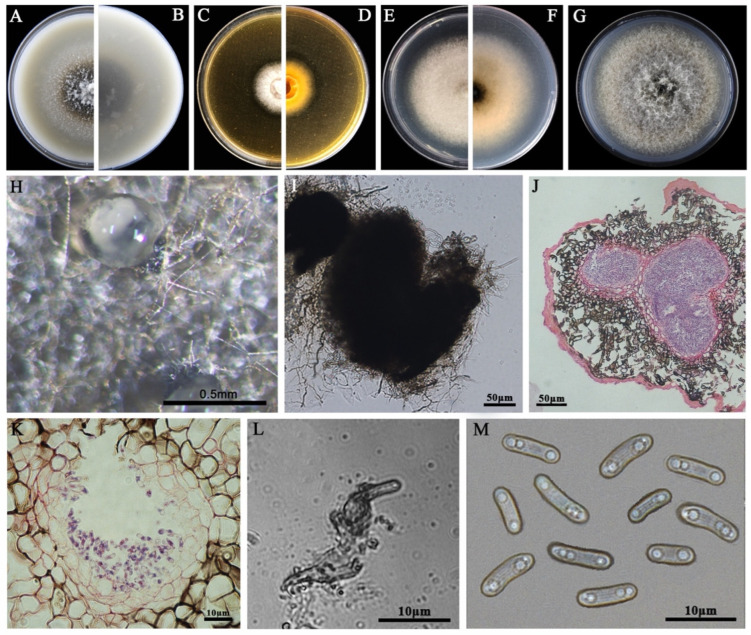
Morphology of *D. macropodii* P5. (**A**,**B**) Colony on OA (front and reverse); (**C**,**D**) colony on MEA (front and reverse); (**E**,**F**) colony on PDA (front and reverse). The cultures in (**A**–**F**) were incubated at 20 °C for 7 days; (**G**) colony on PDA (20 °C, 14 d); (**H**) pycnidia formed on PDA (note the light pycnidiospore ooze on the surface of the pycnidium); (**I**) a crushed pycnidium; (**J**,**K**) sections of pycnidia; (**L**) conidiogenous cells; (**M**) pycnidiospores (conidia).

**Figure 8:**
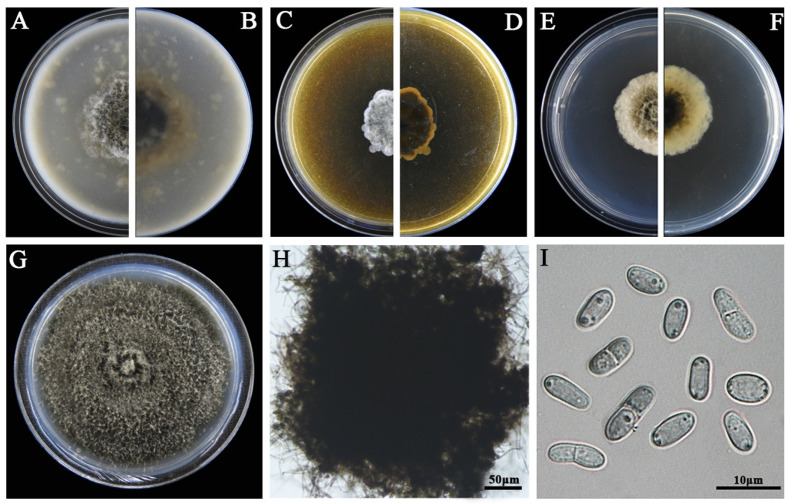
Morphology of *B. exigua* P8. (**A**,**B**) Colony on OA (front and reverse); (**C**,**D**) colony on MEA (front and reverse); (**E**,**F**) colony on PDA (front and reverse). The cultures in (**A**–**F**) were incubated at 20 °C for 7 days; (**G**) colony on PDA (20 °C, 14 d); (**H**) a crushed pycnidium; (**I**) pycnidiospores (conidia).

The eight LbR strains and *L. biglobosa* Lb20 differed in growth rates on MEA, OA, and PDA, and in the morphology of colonies, pycnidia, and pycnidiospores (Table 2). Two LbR strains (P5, P8) and *L. biglobosa* Lb20 grew slowly on at least one of these media, with the average growth rates ranging from 0.7 to 1.8 mm/d on MEA, from 2.0 to 2.4 mm/d on OA, and from 1.5 to 2.3 mm/d on PDA. The remaining six LbR strains (P1, P2, P3, P4, P6, P7) grew rapidly on all of these media, with the average growth rates ranging from 2.2 to 2.6 mm/d on MEA, from 1.7 to 3.8 mm/d on OA, and from 2.2 to 3.9 mm/d on PDA. After incubation for 14 d, the eight LbR strains and Lb20 formed colonies with yellow, brown, or dark brownish substrate mycelia, whitish fluffy aerial mycelia, and black pycnidia (Figure 2, Figure 3, Figure 4, Figure 5, Figure 6, Figure 7 and Figure 8). Cream or pink pycnidiospore oozes (mucilages) were exuded from the pycnidia and accumulated on the top of the pycnidia. Six LbR strains (P1, P3, P4, P5, P6, P7) and Lb20 produced one-celled pycnidiospores of three different shapes: allantoid (P5, P6, P7), cylindroid (P1, P3, Lb20), and ellipsoid (P4). The remaining two LbR strains (P2, P8) produced both one-celled (majority) and double-celled (minority) pycnidiospores of ellipsoidal shape. Moreover, the eight LbR strains and Lb20 differed greatly in the size of pycnidiospores; six LbR strains (P2, P4, P5, P6, P7, P8) produced large pycnidiospores 8.0 to 8.6 μm in length and 2.7 to 4.4 μm in width, and the remaining two LbR strains (P1, P3) and *L. biglobosa* Lb20 produced small pycnidiospores 4.2 to 4.9 μm in length and 1.8 to 2.1 μm in width (Table 2). These morphological characteristics matched the species description for *Boeremia*, *Didymella,* and *Leptosphaeria* [13,33], thus validating the above-mentioned phylogenetic identification of the eight LbR strains as five species of *Didymella* (P1 to P7) and one species of *Boeremia* (P8).

### 3.3. Virulence of the LbRs on Rapeseed

The eight LbR strains differed from *L. biglobosa* Lb20 in their virulence on cotyledons and the stems of rapeseed (Table 3). *L. biglobosa* Lb20 caused a severe infection on cotyledons at 7 dpi and on stems at 21 dpi. The average cotyledon lesion diameter reached 8.2 mm; the average lesion length on the stem epidermis and in the stem pith reached 16.3 and 65.3 mm, respectively. In contrast, the eight LbR strains caused a slight infection; they produced small lesions on cotyledons and stems. The average cotyledon lesion diameters ranged from 1.1 to 1.7 mm (13–21% of that for Lb20), the stem epidermal lesion length ranged from 2.0 to 11.7 mm (12–72% of that for Lb20), and the stem pith lesion length ranged from 2 to 30.8 mm (3–47% of that for Lb20).

### 3.4. The Effect of the LbRs on Infection by L. biglobosa

**Figure 9:**
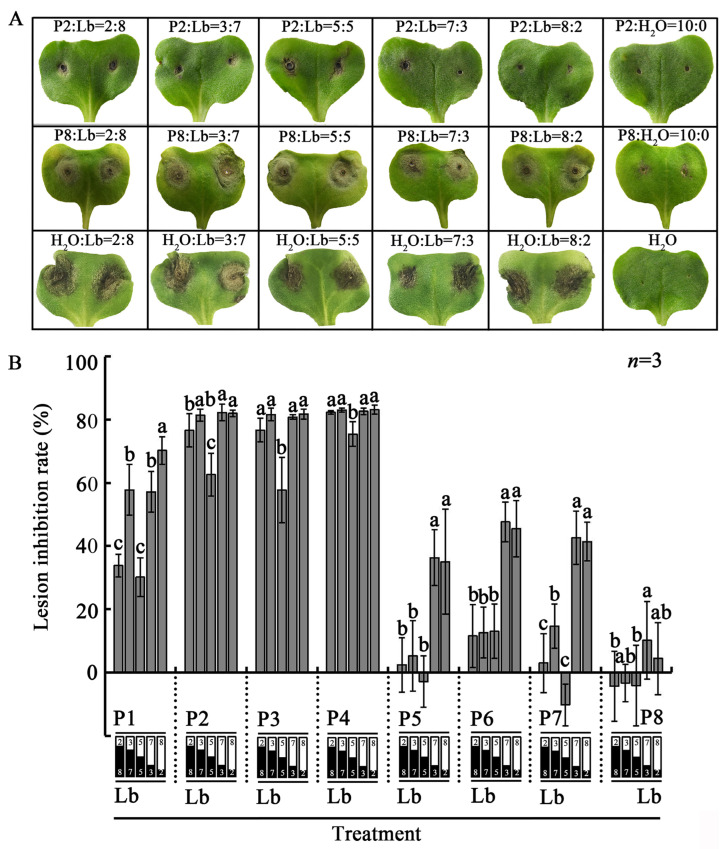
Effects of the LbR strains P1 to P8 on infection of cotyledons of rapeseed by *L. biglobosa* Lb20 (20 °C, 7 d). (**A**) Cotyledons of different treatments showing difference in lesion size; (**B**) histograms showing efficacy of the LbR strains in suppression of infection by *L. biglobosa*. Means ± S.D. labeled with the same letters in each combination of LbR and Lb20 are not significantly different (*p* > 0.05) according to the least significant difference test.

The eight LbR strains showed different effects on infection by *L. biglobosa* Lb20 (Figure 9). Four LbR strains (P1, P2, P3, P4) showed a highly suppressive effect on infection by Lb20; the average lesion diameters in the treatments of P1 + Lb20, P2 Lb20, P3 + Lb20, and P4 + Lb20 at six spore ratios (LbR:Lb20, 2:8, 3:7, 5:5, 7:3, 8:2) were reduced by 30–70%, 63–82%, 58–82%, and 75–83%, respectively, compared to the average lesion diameters caused by Lb20 alone at the corresponding spore concentrations. Three LbR strains (P5, P6, P7) showed a moderately suppressive effect on infection by Lb20; the effect depended on the proportion of their pycnidiospores with Lb20 in the mixed inoculum. In the low-proportion treatments (LbR:Lb20 = 2:8, 3:7, 5:5), the suppressive effect was lower than 15%, whereas in the high-proportion treatments (LbR:Lb20 = 7:3, 8:2), the suppressive effect was increased to 35–48%. The LbR strain P8 had no detectable suppressive effect (<10%) on infection by *L. biglobosa*.

**Figure 10:**
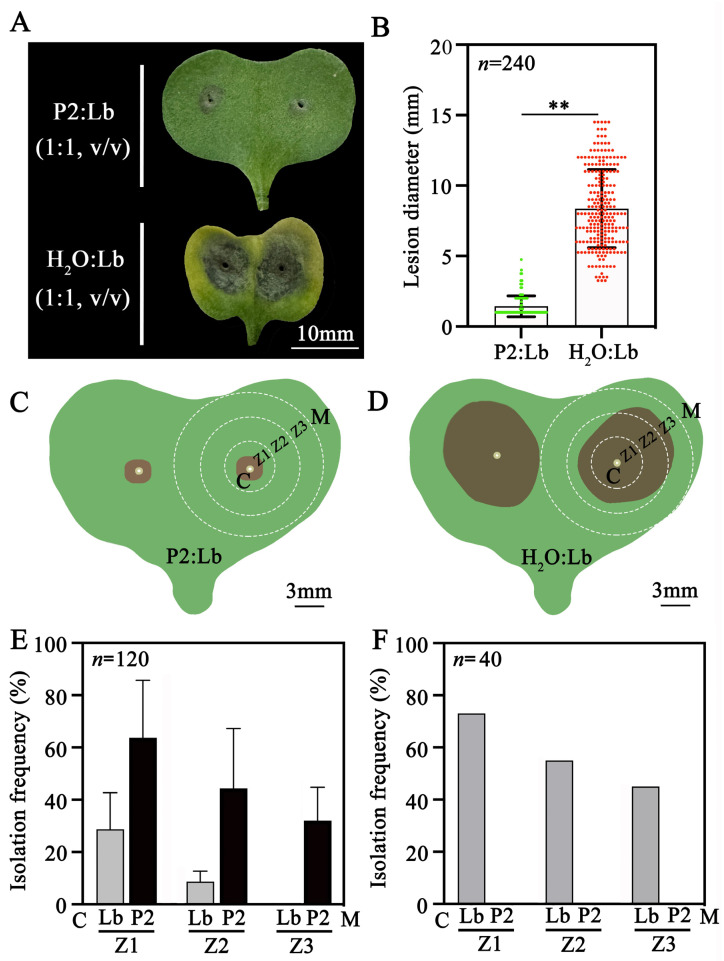
Detection of endophytic colonization of rapeseed cotyledons by *D. macrostoma* P2 and *L. biglobosa* Lb20. (**A**) Cotyledons inoculated with pycnidiospore mixture of P2 and Lb20, and pycnidiospores of Lb20 alone; (**B**) histogram showing lesion diameters in two treatments (data from three trials). ** significantly different at *p* < 0.01 according to Student’s *t* test. (**C**,**D**) Schematic diagrams for sampling rapeseed cotyledon tissues to strain P2 and Lb20; (**E**,**F**) histograms showing isolation frequencies of the two fungi (data from three trials in (**E**) and only one trial in (**F**)).

In the additional experiment, the co-inoculation of *D. macrostoma* P2 and *L. biglobosa* Lb20 (1:1) on rapeseed cotyledons also caused a slight infection with the formation of an average lesion diameter of 1.4 mm; the value was significantly (*p* < 0.01) smaller than that of 8.4 mm caused by *L. biglobosa* Lb20 alone (Figure 10A,B). Internal colonization of cotyledon tissues by P2 and Lb20 was detected in different zones (Z1, Z2, Z3) around the inoculation point (Figure 10C,D and Figure S3). In the treatment of P2 + Lb20, P2 was isolated in Z1, Z2, and Z3 with isolation frequencies of 64%, 44%, and 32%, respectively; Lb20 was isolated in Z1 and Z2 with lower isolation frequencies (29% and 9%, respectively) than those of P2, but was not isolated in Z3 (Figure 10E). In the treatment of Lb20 alone, Lb20 was isolated at frequencies of 73%, 55%, and 45% in Z1, Z2, and Z3, respectively, whereas P2 was not isolated at all in this treatment due to the absence of P2 in the inoculum (Figure 10F). The above results indicated that P2 can colonize and occupy a certain niche in rapeseed cotyledon, which may be a possible mechanism for limiting *L. biglobosa* infection.

### 3.5. The Efficacy of Cultural Filtrates of the LbRs in the Suppression of L. biglobosa Infection

**Figure 11:**
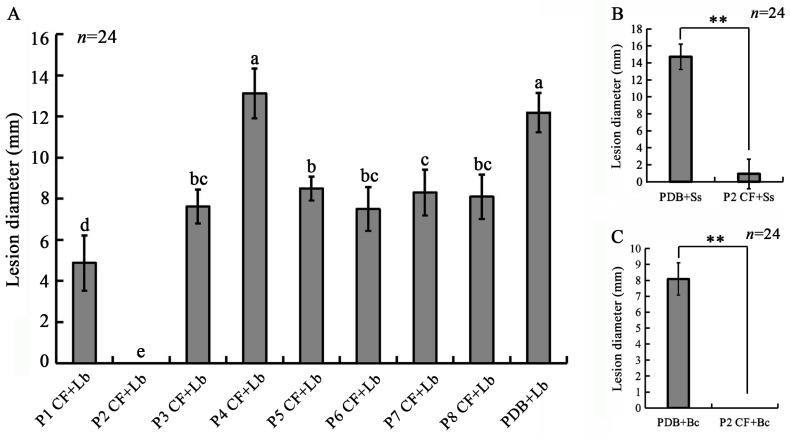
Effects of cultural filtrates of strains P1 to P8 on infection of rapeseed leaves by three rapeseed pathogens. (**A**) Histogram showing leaf lesion diameters caused by *L. biglobosa* (Lb) in different treatments (7 dpi, 20 °C). CFs, cultural filtrates; PDB, potato dextrose broth. Means ± S.D. labeled with the same letters are not significantly different (*p* > 0.05) according to least significant difference test; (**B**,**C**) histograms showing leaf lesion diameters caused by *S. sclerotiorum* (Ss) and *B. cinerea* (Bc), respectively (20 °C, 3 dpi). ** significantly different at *p* < 0.01 according to Student’s *t* test.

The cultural filtrates (CFs) of the eight LbR strains differed in efficacy in the suppression of infection by *L. biglobosa* Lb20 on rapeseed leaves (Figure 11A). In the control treatment without a CF, a severe infection was observed, and the average lesion diameter reached 12 mm. However, in the treatments with the CF of seven LbR strains (P1, P2, P3, P5, P6, P7, P8), no visible infection or reduced infection was observed. The suppressive efficacy (e.g., reduction in lesion diameter compared to the control treatment) ranged from 30% by P5 CF in P5 CF + Lb20 to 100% by P2 CF in P2 CF + Lb20. In contrast, the P4 CF showed no detectable efficacy in the suppression of *L. biglobosa* infection; the average lesion diameter reached 13 mm in this treatment (Figure 11A).

Moreover, P2 CF showed high efficacy in the suppression of infection by *B. cinerea* and *S. sclerotiorum* on rapeseed leaves. In the control treatments, both *B. cinerea* and *S. sclerotiorum* aggressively colonized rapeseed leaves, causing necrotic lesions at 3 dpi with average lesion diameters of 8 and 15 mm by *B. cinerea* and *S. sclerotiorum*, respectively (Figure 11B,C). However, no visible infection was observed in the treatments with P2 CF, and a slight infection was observed when inoculated with Ss-1 with the formation of tiny lesions 1 mm in diameter. The results also showed that the color of the rapeseed leaves treated with P2 CF and water alone appeared green (Figure S4), suggesting that P2 CF may have no phytotoxicity towards rapeseed leaves.

### 3.6. The Antifungal Activity of P2 CF and P2 PS

**Figure 12:**
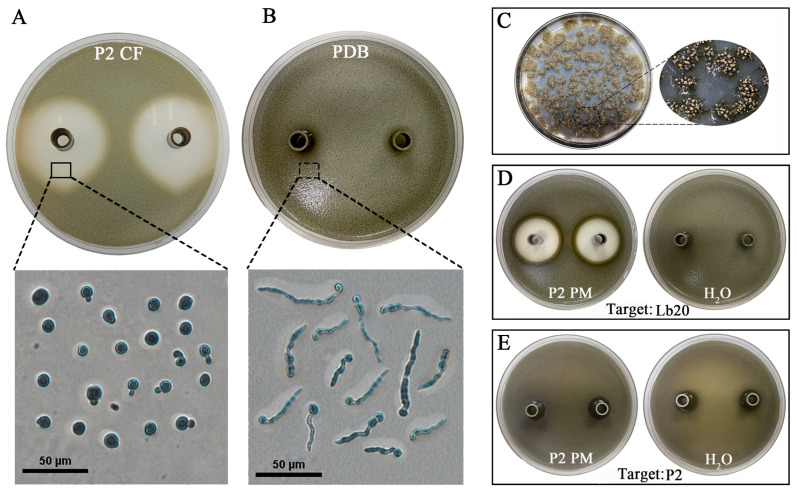
Specific antifungal activity of *D. macrostoma* P2 to *L. biglobosa* in the agar diffusion assay (20 °C, 4 d). (**A**) Two clear zones around two Oxford cups containing P2 CFs; note inhibition of *L. biglobosa* spore germination and germ tube elongation. (**B**) No clear zone formation around two Oxford cups containing fresh PDB; note normal pycnidiospore germination of *L. biglobosa* and germ tube elongation. (**C**) PDA culture of P2 with sticky pycnidiospore mucilages exuded from pycnidia; the mucilages were washed off with water for preparation of a pycnidiospore suspension, and pycnidiospore-associated substances (e.g., P2 PSs) were dissolved in the pycnidiospore suspension. (**D**) Two clear zones caused by P2 PSs and no clear zones caused by water on PDA containing pycnidiospores of *L. biglobosa*; (**E**) no clear zones caused by P2 PSs and water on PDA containing pycnidiospores of *D. macrostoma* P2.

In the agar diffusion assay, P2 CF in Oxford cups diffused into PDA containing pycnidiospores of *L. biglobosa* Lb20 and caused the formation of inhibition zones (e.g., clear zones) around the Oxford cups due to the inhibition of germination of pycnidiospores and the elongation of term tubes (Figure 12A), whereas fresh PDB in the Oxford cups failed to form inhibition zones due to normal pycnidiospore germination and germ tube elongation (Figure 12B). The results also showed that P2 PS (e.g., pycnidiospore-associated substances, Figure 12C) in the Oxford cups caused the formation of inhibition zones on PDA also containing pycnidiospores of *L biglobosa* Lb20 (Figure 12D), indicating antifungal activity towards *L. biglobosa*. However, P2 PS failed to produce inhibition zones on the P2 pycnidiospores added PDA (Figure 12E), indicating no antifungal activity towards P2 itself.

**Figure 13:**
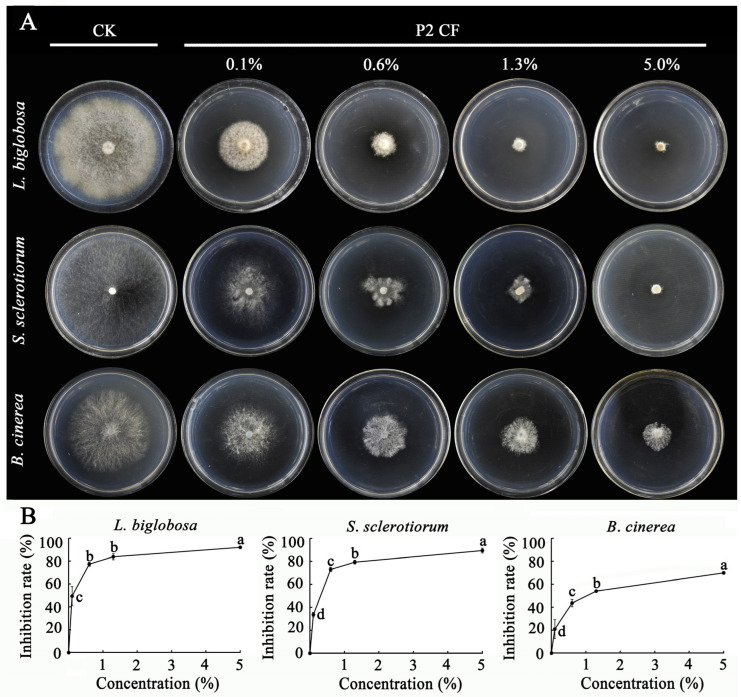
Inhibition of fungal mycelial growth by cultural filtrates (CFs) of *D. macrostoma* P2 in an agar amendment assay. (**A**) Fungal colonies on PDA alone and in PDA with P2 CFs added at different concentrations. (**B**) Inhibition rates against the three pathogens. Means ± S.D. in each figure labeled with the same letter are not significantly different (*p* > 0.05) according to least significant difference test.

In the agar amendment bioassay, the P2 CF added in PDA at 0.1% to 5.0% (*v*/*v*) significantly (*p* < 0.05) reduced the colony size of *L. biglobosa*, *S. sclerotiorum,* and *B. cinerea*, compared to the colony size of these fungi on PDA without P2 CF alone (Figure 13A). With the increase in the concentration of P2 CFs from 0.1% to 5.0%, the inhibition rates (e.g., the percentage of colony size reduction) were consistently increased from 49% to 92% for *L. biglobosa*, from 33% to 89% for *S. sclerotiorum*, and from 20% to 70% for *B. cinerea* (Figure 13B).

### 3.7. Penicillither Yield and Antifungal Activity

**Figure 14:**
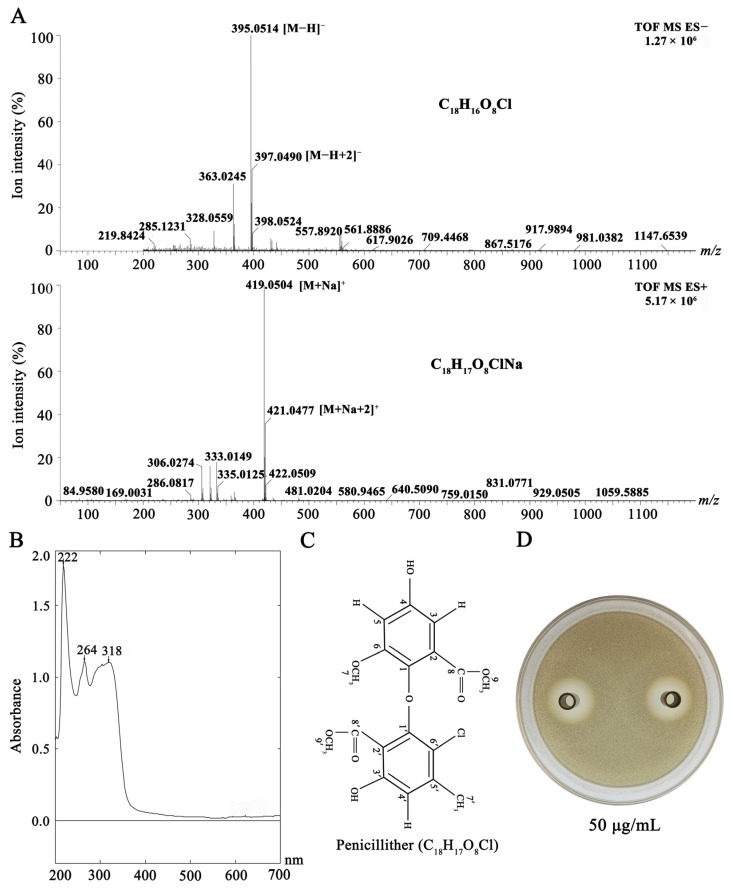
Identification of penicillither produced by *D. macrostoma* P2. (**A**) LC-MS spectra in negative and positive modes for calculation of molecular mass and inference of molecular formula; (**B**) UV-Vis spectrum; (**C**) chemical structure; and (**D**) antifungal activity against *L. biglobosa*.

The purified fraction Fr.4.4 was a light-yellow amorphous powder (Figure S1), and it was soluble in methanol and DMSO. LC-MS detection showed that the peak intensity ratio at *m/z* 395 [M-H]^−^ and 397 [M-H+2]^−^ was approximately 3:1, and this ratio was also observed at *m/z* 419 [M+Na]^+^ and 421 [M+Na+2]^+^. These results indicate that there is a chlorine atom in Fr.4.4 (Figure 14A) [34]. The molecular formula of Fr.4.4 was thus deduced to be C_18_H_17_O_8_Cl based on the positive HR ESI-MS spectrum (*m/z*: observed 419.0503 [M+Na]^+^, calculated 419.0510) and the negative HR ESI-MS spectrum (*m/z*: observed 395.0517 [M-H]^−^, calculated 395.0534). CAS SciFinder^®^ (https://origin-scifinder.cas.org (accessed on 28 November 2023)) indicates that Fr.4.4 might belong to penicillither or methyl 3-chloroasterric acid. UV absorption determination indicated that Fr.4.4 formed three characteristic absorption peaks at 222, 264, and 318 nm (Figure 14B), suggesting that this compound is penicillither, and this was confirmed by the partial data of the ^1^H-NMR (DSMO, 600 MHz) and ^13^C NMR (DSMO, 125 MHz) spectra (Table 4; Figures S5 and S6). Quantitative analysis indicated that the content of penicillither was 45.5% in P2 CE, and the concentration of penicillither was 127.3 ± 18.6 μg/mL in P2 CF and 117.8 ± 21.5 μg/mL in P2 PS (1 × 10^8^ spores/mL). The agar diffusion bioassay showed that penicillither had antifungal activity towards *L. biglobosa* (Figure 14C), and it produced inhibition diameters of 1.8 and 2.3 cm at concentrations of 50 and 100 μg/mL, respectively.

## 4. Discussion

The present study clarified the taxonomic status of the eight LbR strains (P1 to P8). They belong to the same family, namely, *Didymellaceae*. Seven LbR strains belong to five species of *Didymella*, including two unidentified species of *Didymella* (P1, P3), *D. macrostoma* (P2), *D. glomerata* (P4), and *D. macropodii* (P5, P6, P7). The remaining one, namely strain P8, belongs to *Boeremia exigua*. All of these didymellaceous species had the telemorphs of *Phoma* [33]. Molecular phylogeny suggests that the LbR strains are distantly related to *L. biglobosa* (anamorph: *Plenodomus biglobosus*), which belongs to the family *Leptosphaeriaceae*. Strains P1 and P3 are closely related to *D. bellidis*; they formed two unique branches, suggesting that they might belong to two novel species. A definition of these two taxa as novel species of *Didymella* will be finalized in the future when more strains are collected.

*D. macrostoma*, *D. glomerata*, *D. macropodii,* and *B. exigua* can be plant pathogens [13]. For example, *D. macrostoma* can cause leaf spot on *Pinckneya pubens* [36], leaf chlorosis and necrosis on *Cirsium arvense* [37], and root and crown disease on *Lepidium draba* [38]. *D. glomerata* can cause leaf blight and twig canker on *Pyrus communis* [39], as well as leaf blight on *Pistacia vera* [40]; *D. macropodii* can cause hypocotyl discoloration and constriction on *Brassica napus* [41]; and *B. exigua* var. *exigua* can cause black (tan) spot on *Glycine max* [42], *Phaseolus lunatus* [43], and *Pisum sativum* [44], as well as post-harvest fruit rot on *Malus × domestica* [45]. Recently, *B. exigua* var. *exigua* was reported to occur in China, causing branch blight on *Juglans sinensis* [46]. However, the eight LbR strains obtained in this study are probably not the pathogens or at least not the important pathogens of oilseed rape. First, the LbR strains occurred at the frequency of 0.1% (8 strains in 6023 strains; Deng et al., 2023); the value was much lower than that of 99.9% for *L. biglobosa* in the same ecological niches as the LbR strains. Second, the LbR strains showed significantly (*p* < 0.05) lower virulence than *L. biglobosa*. Therefore, they are probably occasional intruders on stems readily colonized by *L. biglobosa*. Moreover, previous studies indicated that *D. macrostoma* and *D. glomerata* can be endophytes living in plant tissues without causing visible disease symptoms [47,48]. In this study, *D. macrostoma* P2 and *D. glomerata* P4 caused negligible infection both in cotyledons and in the stems of oilseed rape. This study found that *D. macrostoma* P2 was consistently isolated from healthy cotyledon tissues distant from the inoculation points of *D. macrostoma* P2 and *L. biglobosa* Lb20. This result suggests that *D. macrostoma* P2 is an endophyte of rapeseed. Further studies using visible markers (e.g., green fluorescent protein) to probe the endophytic colonization of *D. macrostoma* P2 in tissues of rapeseed are warranted.

Meanwhile, the taxonomic position of 15 strains of *L. biglobosa* was also clarified based on molecular phylogenies with the DNA sequences of ITS, LSU, *tub2,* and *rpb2*, and they all belong to *L. biglobosa* (anamorph: *Plenodomus*) [49]. Phylogenetic analysis with the DNA sequences of ITS and LSU also showed that the 15 strains of *L. biglobosa* are more closely related to *L. biglobosa* ‘brassicae’. This subspecies is distributed widely in the south of China, where the winter-type oilseed rape is widely planted [50,51]. Recently, Luo et al. (2021) and Deng et al. (2023) reported that *L. biglobosa* ‘canadensis’ is the dominant and widespread blackleg pathogen of rapeseed in the north of China [23,52], where the spring-type rapeseed is widely planted. The reasons for the differential distribution of *L. biglobosa* ‘brassicae’ and *L. biglobosa* ‘canadensis’ in China remain unknown and need further investigation.

The present study demonstrated that *D. macrostoma* P2 significantly suppressed infection by *L. biglobosa* on rapeseed cotyledons when it was co-inoculated with *L. biglobosa*. Two mechanisms might be involved in the suppression of *L. biglobosa* infection by *D. macrostoma* P2. One is the production of antifungal metabolites in the pycnidiospores of *D. macrostoma* P2, and the other is the endophytic colonization of rapeseed plant tissues by *D. macrostoma* P2. We found that the P2 PSs (pycnidiospore-associated substances) had inhibitory activity on *L. biglobosa*. The inhibition might occur in the inoculation points with *D. macrostoma* P2 and *L. biglobosa* Lb20. Previous studies showed that *L. biglobosa* can conduct endophytic growth in rapeseed tissues during the period of latent infection [53,54]. However, *D. macrostoma* displayed more aggressiveness than *L. biglobosa* in the endophytic colonization of cotyledons of rapeseed, thereby generating suppression against infection by *L. biglobosa*. Previous studies have demonstrated that endophytic fungi could play a beneficial role in plants under certain circumstances [55,56,57]. For example, non-pathogenic endophytic strains of *Fusarium oxysporum* could yield a biocontrol effect on infection by pathogenic *F. oxysporum* [58]. Additional studies are necessary to investigate the mechanisms of enphytic growth of *D. macrostoma* P2 and to evaluate the biocontrol effects of the enphytic growth of *D. macrostoma* P2 on rapeseed diseases, including clubroot (*Plasmodiophora brassicae*), sclerotinia stem rot (*S. sclerotiorum*), and blackleg (*L. biglobosa*).

In addition to inhibiting *L. biglobosa*, the cultural filtrates (CFs) of *D. macrostoma* P2 showed high efficacy in the suppression of *B. cinerea* and *S. sclerotiorum* on rapeseed leaves. These results suggest that *D. macrostoma* P2 and the antifungal metabolites produced by this fungus have a wide inhibitory spectrum. Previous studies by Aire et al. [17,18] showed that *D. glomerata* (a close relative of *D. macrostoma*) is an effective biocontrol agent against rapeseed clubroot through the production of epoxydon. *D. macrostoma* has been tested to control broadleaf weeds such as Canadian thistle (*Cirsium arvense*) [19,59], and strain 94–44B of *D. macrostoma* has been registered as a bioherbicide in Canada and the USA [37]. This study showed that the metabolites of *D. macrostoma* P2 had no detectable phytotoxicity towards leaves of rapeseed. Therefore, the antifungal metabolites of *D. macrostoma* P2 can be exploited to control *B. cinerea* and *S. sclerotiorum*. Additional studies are necessary to determine the efficacy of the antifungal metabolites produced by these particular strains of *D. macrostoma* in the suppression of rapeseed diseases under field conditions.

Previous studies have reported several bioactive metabolites produced by *D. macrostoma*, including macrocidins (e.g., cyclic tetramic acids) with herbicidal activity [19,59], oxazole carboxylic acids with antibacterial and anti-tumor activities [60], as well as mono-(2-ethylhexyl) phthalate with antibacterial and antifungal activities [47]. In this study, we found that the application of P2 CFs on rapeseed leaves effectively suppressed infection by *L. biglobosa*, *B. cinerea,* and *S. sclerotiorum*. However, it did not cause any visible phytotoxicity towards rapeseed leaves, suggesting that *D. macrostoma* P2 may have no capability to synthesize macrocidins. Meanwhile, we identified an antifungal metabolite, namely penicillither, in PDB cultures and pycnidiospore suspensions of *D. macrostoma* P2. Penicillither was first isolated and identified in *Penicillium* PSU-RSPG99 [61] and in later studies on *Aspergillus capensis* and *A*. *flaviceps* [35,62]. Penicillither has a diphenyl ether skeleton structure, and its benzene ring contains a chlorine atom, which is a chlorinated diphenyl ether compound. It is also a homologue of asterric acid, which is diverse in structure and biological activity. In 1960, asterric acid was first isolated from the fermentation broth of *Aspergillus terreus* [63]. In addition, Jayasuriya et al. (1995) identified a variety of asterric acid analogues from the fermentation broth of *Phoma* sp., two of which had inhibitory activity against the new tetrapeptide chalchol-invertase (FPTase) [64]. In future studies, it is necessary to sequence the whole genome of *D. macrostoma* P2, which can be used to mine the genes for the biosynthesis of the secondary metabolites in that fungus.

## 5. Conclusions

This research demonstrated that *Didymella macrostoma* P2 is a broad-spectrum antimicrobial agent that can be exploited to control rapeseed diseases caused by *L. biglobosa*, *B. cinerea,* and *S. sclerotiorum*. *D. macrostoma* P2 is a novel producer of penicillither, which was detected both in PDB cultures and in pycnidiospore mucilages. Moreover, this study found that *D. macrostoma* P2 could possibly restrict *L. biglobosa* infection in rapeseed cotyledons through the production of penicillither and the aggressive endophytic occupation of the ecological niche. At present, the endophytic mechanism of *D. macrostoma* P2 remains totally unknown and needs further investigation, as this can improve the applicability of using *D. macrostoma* P2 to control rapeseed disease. Overall, this study provides a case study of screening pathogen-associated fungi as biocontrol agents.

## Figures and Tables

**Table 1:** Fungal strains from rapeseed, collection place, and year, as well as GenBank accession numbers for ITS, LSU, *tub2*, and *rpb2*.

Species and Strain	Collection Place	GenBank Accession Number			
		ITS1 ^1^	LSU	*tub2*	*rpb2*
*L. biglobosa* Lb20	Wuhan, Hubei Province, China, 2017	MT807903	MT611125	MT807904	MT807905
*L. biglobosa* Lb982	Anshun, Guizhou Province, China, 2018	MT611111	MT611126	MT650708	MT683499
*L. biglobosa* Lb983	Anshun, Guizhou Province, China, 2018	MT611112	MT611127	MT650709	MT683500
*L. biglobosa* Lb1024	Bijie, Guizhou Province, China, 2018	MT611113	MT611128	MT650710	MT683501
*L. biglobosa* Lb1025	Bijie, Guizhou Province, China, 2018	MT611114	MT611129	MT650711	MT683502
*L. biglobosa* Lb1041	Bijie, Guizhou Province, China, 2018	MT611115	MT611130	MT650712	MT683503
*L. biglobosa* Lb3974	Longnan, Gansu Province, China, 2019	MT611116	MT611131	MT650713	MT683504
*L. biglobosa* Lb3995	Longnan, Gansu Province, China, 2019	MT611117	MT611132	MT650714	MT683505
*L. biglobosa* Lb4055	Longnan, Gansu Province, China, 2019	MT611118	MT611133	MT650715	MT683506
*L. biglobosa* Lb4068	Longnan, Gansu Province, China, 2019	MT611119	MT611134	MT650716	MT683507
*L. biglobosa* Lb4079	Longnan, Gansu Province, China, 2019	MT611120	MT611135	MT650717	MT683508
*L. biglobosa* Lb4099	Longnan, Gansu Province, China, 2019	MT611121	MT611136	MT650718	MT683509
*L. biglobosa* Lb4149	Tianshui, Gansu Province, China, 2019	MT611122	MT611137	MT650719	MT683510
*L. biglobosa* Lb4150	Tianshui, Gansu Province, China, 2019	MT611123	MT611138	MT650720	MT683511
*L. biglobosa* Lb4152	Tianshui, Gansu Province, China, 2019	MT611124	MT611139	MT650721	MT683512
*Didymella* sp. P1	Enshi, Hubei Province, China, 2017	MK121680	MK121684	MK160142	MK127941
*D. macrostoma* P2	Yunxi, Hubei Province, China, 2017	MK121681	MK121685	MK160143	MK127942
*Didymella* sp. P3	Yunxi, Hubei Province, China, 2017	MK121682	MK121686	MK160144	MK127943
*D. glomerata* P4	Huzhu, Qinghai Province, China, 2017	MK121683	MK121687	MK160145	MK127944
*D. macropodii* P5	Huzhu, Qinghai Province, China, 2018	MK579368	MK579371	MK579377	MK579374
*D. macropodii* P6	Huzhu, Qinghai Province, China, 2018	MK579369	MK579372	MK579378	MK579375
*D. macropodii* P7	Huzhu, Qinghai Province, China, 2018	MK579370	MK579373	MK579379	MK579376
*Boeremia exigua* P8	Lincang, Yunnan Province, China, 2019	MT505299	MT505300	MT505301	MT505302

^1^ Abbreviations: ITS, internal transcribed spacer; LSU, 28S ribosomal large subunit rDNA gene; *tub2*, the β-tublin gene; *rpb2*, the gene coding for DNA-dependent RNA polymerase II second largest subunit.

**Table 2:** Mycelial growth rates (20 °C) on three agar media as well as shape and size of the pycnidiospores produced by different fungal strains.

Strain	Mycelial Growth Rate (mm/d)				Pycnidiospores	
	Malt Extract Agar	Oatmeal Agar	Potato Dextrose Agar		Shape	Size in μm (*n* = 50)
*Leptosphaeria biglobosa* Lb20	0.7 ± 0.2 d ^1^	2.4 ± 0.4 c	2.3 ± 0.1 b		Cylindroid	4.6–5.3 (4.9) × 2.0–2.3 (2.1)
*Didymella* sp. P1	2.2 ± 0.4 ab	1.7 ± 0.1 d	2.8 ± 0.3 b		Cylindroid	4.7–5.3 (4.9) × 2.0–2.3 (2.1)
*Didymella macrostoma* P2	2.6 ± 0.1 a	3.5 ± 0.7 a	2.7 ± 0.5 b		Ellipsoid	7.3–9.3 (8.1) × 3.3–4.0 (3.6)
*Didymella* sp. P3	2.2 ± 0.5 ab	2.9 ± 0.3 b	2.2 ± 0.5 b		Cylindroid	4.0–4.7 (4.2) × 1.7–2.0 (1.8)
*Didymella glomerata* P4	2.6 ± 0.3 a	3.4 ± 0.3 a	3.5 ± 0.7 a		Ellipsoid	7.3–8.7 (8.1) × 4.0–4.7 (4.4)
*Didymella macropodii* P5	1.8 ± 0.1 b	2.3 ± 0.3 c	1.6 ± 0.6 c		Allantoid	7.2–9.6 (8.4) × 2.4–2.8 (2.6)
*Didymella macropodii* P6	2.5 ± 0.6 a	2.3 ± 0.4 c	3.5 ± 0.9 a		Allantoid	7.6–10.4 (8.6) × 2.8–3.5 (3.1)
*Didymella macropodii* P7	2.6 ± 0.6 a	3.8 ± 0.4 a	3.9 ± 0.2 a		Allantoid	6.3–11.1 (8.4) × 2.1–3.5 (2.7)
*Boremia exigua* P8	1.2 ± 0.4 c	2.0 ± 0.4 cd	1.5 ± 0.2 c		Ellipsoid	6.9–9.7 (8.0) × 3.5–5.2 (4.4)

^1^ Mean values for each parameter within each column with the same letters indicate no significant difference according to the least significance test at *p* > 0.05.

**Table 3:** Pathogenicity of *L. biglobosa*, *Didymella* spp., and *Boeremia exigua* P8 on rapeseed (*Brassica napus* cultivar Zhongshuang No.9).

Strain	Cotyledon Lesion Diameter (mm)		Lesions on Stem Epidermis			Lesion in Stem Pith	
			Length (mm)	Width (mm)		Length (mm)	Width (mm)
*Leptosphaeria biglobosa* Lb20	8.2 ± 1.1 a ^1^		16.3 ± 2.6 a	3.8 ± 0.8 a		65.3 ± 6.6 a	7.5 ± 0.8 a
*Didymella* sp. P1	1.1 ± 0.1 b		2.0 ± 0.0 f	1.0 ± 0.0 d		2.0 ± 0.0 e	1.0 ± 0.0 c
*Didymella macrostoma* P2	1.2 ± 0.1 b		8.7 ± 0.8 cd	2.7 ± 0.5 bc		5.0 ± 0.9 e	1.5 ± 0.5 c
*Didymella* sp. P3	1.2 ± 0.1 b		9.2 ± 1.3 c	2.5 ± 0.5 bc		6.5 ± 0.5 e	1.8 ± 0.8 c
*Didymella glomerata* P4	1.3 ± 0.1 b		11.7 ± 4.1 b	2.8 ± 0.4 b		12.0 ± 2.4 d	1.8 ± 0.8 c
*Didymella macropodii* P5	1.6 ± 0.3 b		10.2 ± 1.7 bc	2.8 ± 0.8 b		30.8 ± 2.8 b	6.2 ± 0.8 b
*Didymella macropodii* P6	1.4 ± 0.4 b		9.3 ± 2.3 bc	2.8 ± 1.0 b		26.7 ± 4.1 b	6.3 ± 0.8 b
*Didymella macropodii* P7	1.5 ± 0.3 b		6.7 ± 0.8 de	1.8 ± 0.8 c		27.2 ± 3.8 b	7.0 ± 1.1 ab
*Boeremia exigua* P8	1.7 ± 0.3 b		6.0 ± 0.6 e	2.3 ± 0.8 c		20.5 ± 7.0 c	7.0 ± 0.6 ab

^1^ Mean values for each parameter within each column with the same letters indicate no significant difference according to the least significance test at *p* > 0.05.

**Table 4:** Partial ^1^H and ^13^C NMR spectra of penicillither produced by *D. macrostoma* P2.

Position	*δ*_H_ (m, *J* in Hz) 600 MHz			*δ*_C_	
	This Study	[35]		This Study	[35]
1				138.5	135.4
2				125.7	124.4
3	6.72, d	6.73, d		110.6	110.5
4				ND	150.6
5	6.65, d	6.65, d		104.8	104.7
6				ND	152.9
7				56.6	56.1
8				ND	164.5
9				51.9	51.6
1′				ND	153.4
2′				107.7	107.3
3′				ND	154.5
4′	6.51, s	6.51, s		ND	110.8
5′				141.5	137.9
6′				ND	113.5
7′	2.27, s	2.30, s		20.5	20.2
8′				167.3	164.6
9′				ND	51.8
3′-OH	8.40, s	9.95, br s			
7-OCH_3_	3.22, s	3.23, s			
9-OCH_3_	3.62, s	3.63 s			
9′-OCH_3_	3.61, s	3.60, s			

ND, not detectable due to weak signals.

## Data Availability

Data is contained within the article and Supplementary Material.

## Supplementary Materials

The following supporting information can be downloaded at: https://www.mdpi.com/article/10.3390/jof9121167/s1, Figure S1: The procedure for purification of the fraction of Fr. 4.4 and determination of purity of the sub-fraction Fr.4.4.; Figure S2: A Bayesian phylogenetic tree of 38 fungal taxa.The tree was constructed using concatenated sequences of ITS and LSU; Figure S3: Isolation of *D. macrostoma* P2 and *L. biglobosa* Lb20 in rapeseed cotyledons; Figure S4: Two leaves of rapeseed treated with the culture filtrate (CF) of *D. macrostoma* P2 and PDB alone; Figure S5: ^13^C NMR (125 MHz, DSMOMeOH-d_6_, NMR) spectrum for penicillither from *Didymella macrostoma* P2; Figure S6: ^1^H NMR (600 MHz, DSMO-d_6_, NMR) spectrum for penicillither A from *Didymella macrostoma* P2; Table S1: PCR primers used in this study for amplification of different genes; Table S2: PCR thermal programs for amplification of different fungal genes; Table S3: Origin of the fungal isolates used in this study and GenBank accession numbers; Refs [65,66,67,68,69] are cited in Supplementary Materials.
