# Supplementary material for: The Identity, Virulence, and Antifungal Effects of the Didymellacesous Fungi Associated with the Rapeseed Blackleg Pathogen Leptosphaeria biglobosa
Source: J Fungi (Basel). 2023 Dec 4;9(12):1167. doi: 10.3390/jof9121167 (PMC10744798; doi:10.3390/jof9121167)
Supplement: Supplementary file 1 [file jof-09-01167-s001.zip › jof-2726772-supplementary.pdf]

Supplementary Materials

The Identity, Virulence and Antifungal Effects of the Didymellacesous Fungi Associated with the Rapeseed Blackleg Pathogen *Leptosphaeria biglobosa*

Junyu Cheng [1], Tao Luo [1], Mingde Wu [1], Long Yang [1], Weidong Chen [2], Guoqing Li [1] and Jing Zhang [1]*

[1] State Key Laboratory of Agricultural Microbiology and Key Laboratory of Plant Pathology of Hubei Province, Huazhong Agricultural University, Wuhan 430070, China;

[2] United States Department of Agriculture, Agricultural Research Service, Washington State University, Pullman, WA, 99164, USA.

*Correspondence: author: Dr. J. Zhang, E-mail: zhangjing1007@mail.hzau.edu.cn

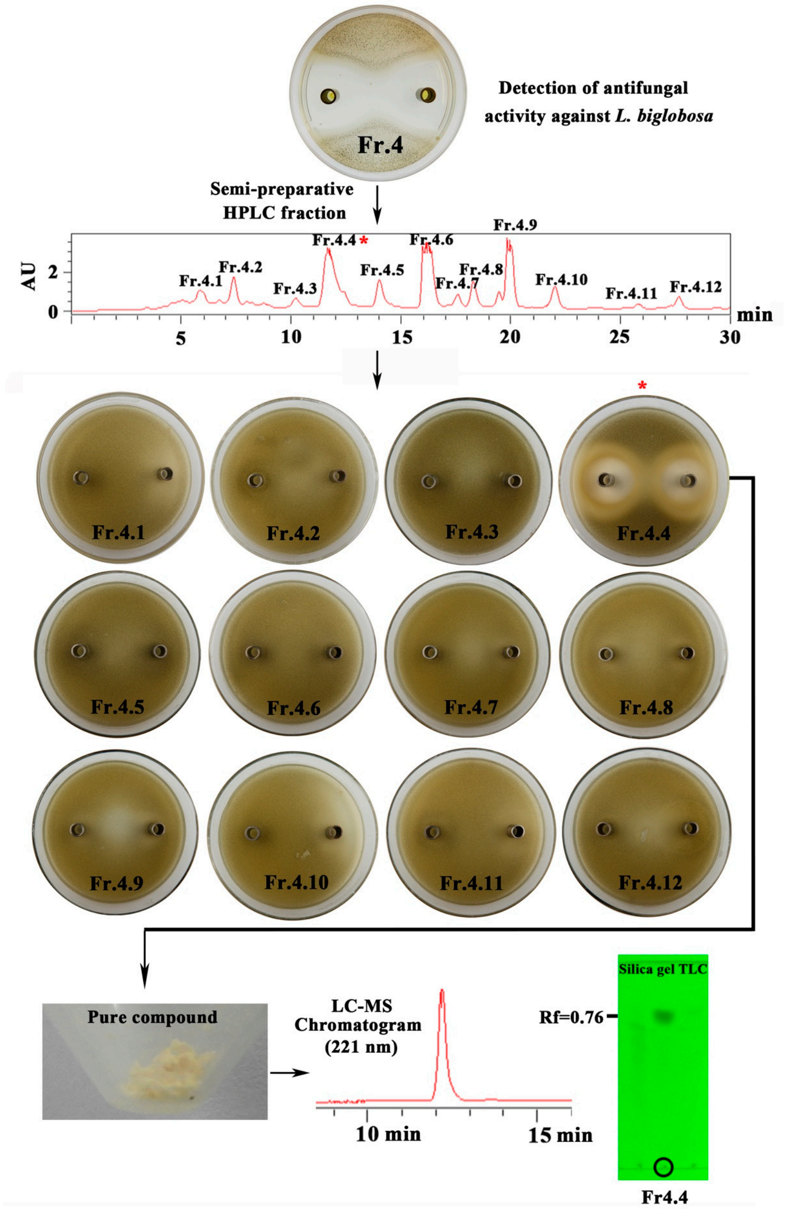

Figure S1. The procedure for purification of the fraction of Fr. 4.4 and determination of purity of the sub-fraction Fr.4.4.

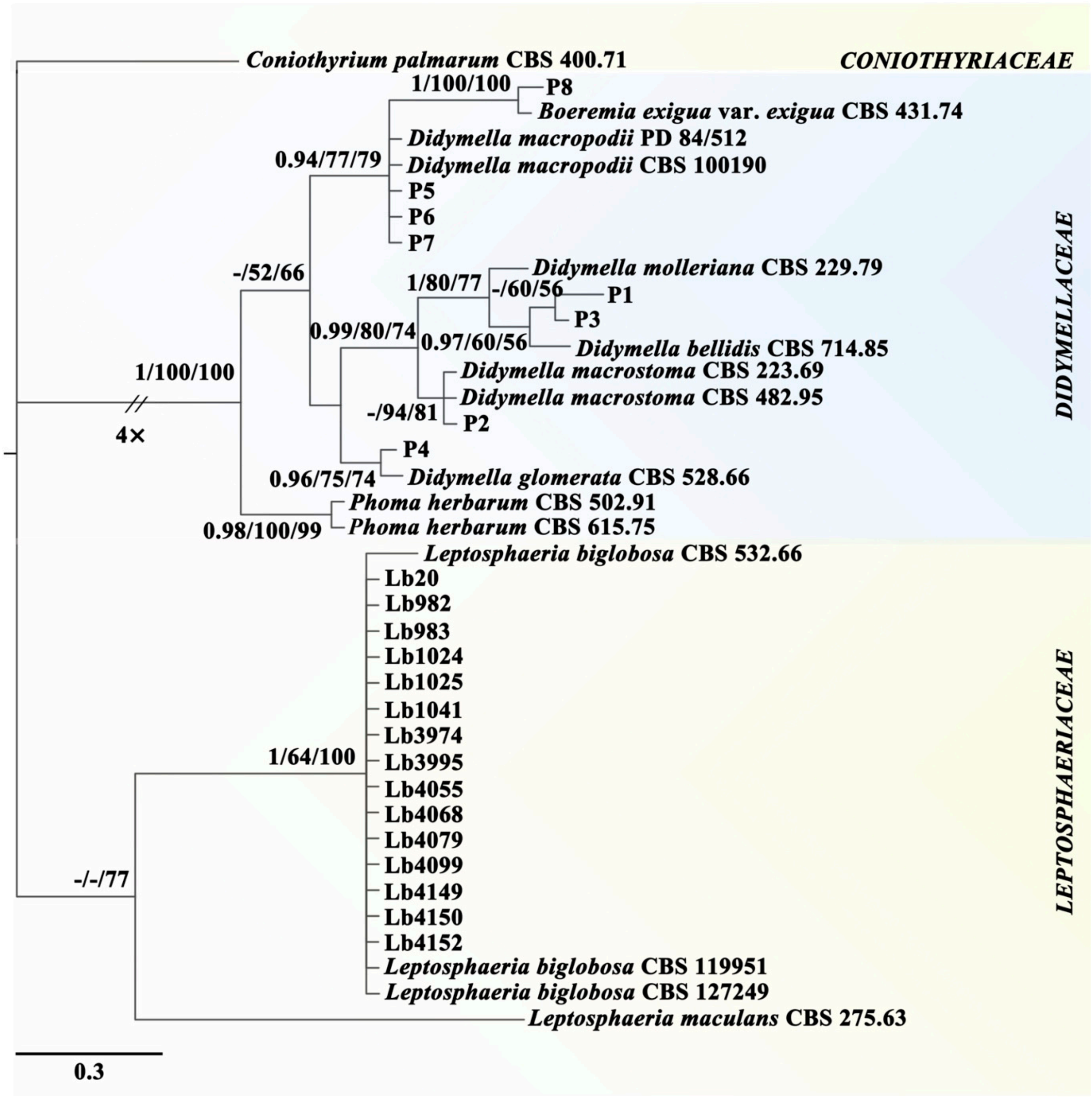

Figure S2. A Bayesian phylogenetic tree of 38 fungal taxa.The tree was constructed using concatenated sequences of ITS and LSU. Bayesian posterior probability (PP ≥ 0.90), RAxML bootstrap support values (ML ≥ 50%) and MP bootstrap support values (MP ≥ 50%) were shown at the nodes (PP/ML/MP). Scale bar indicates 0.3% sequence divergence.

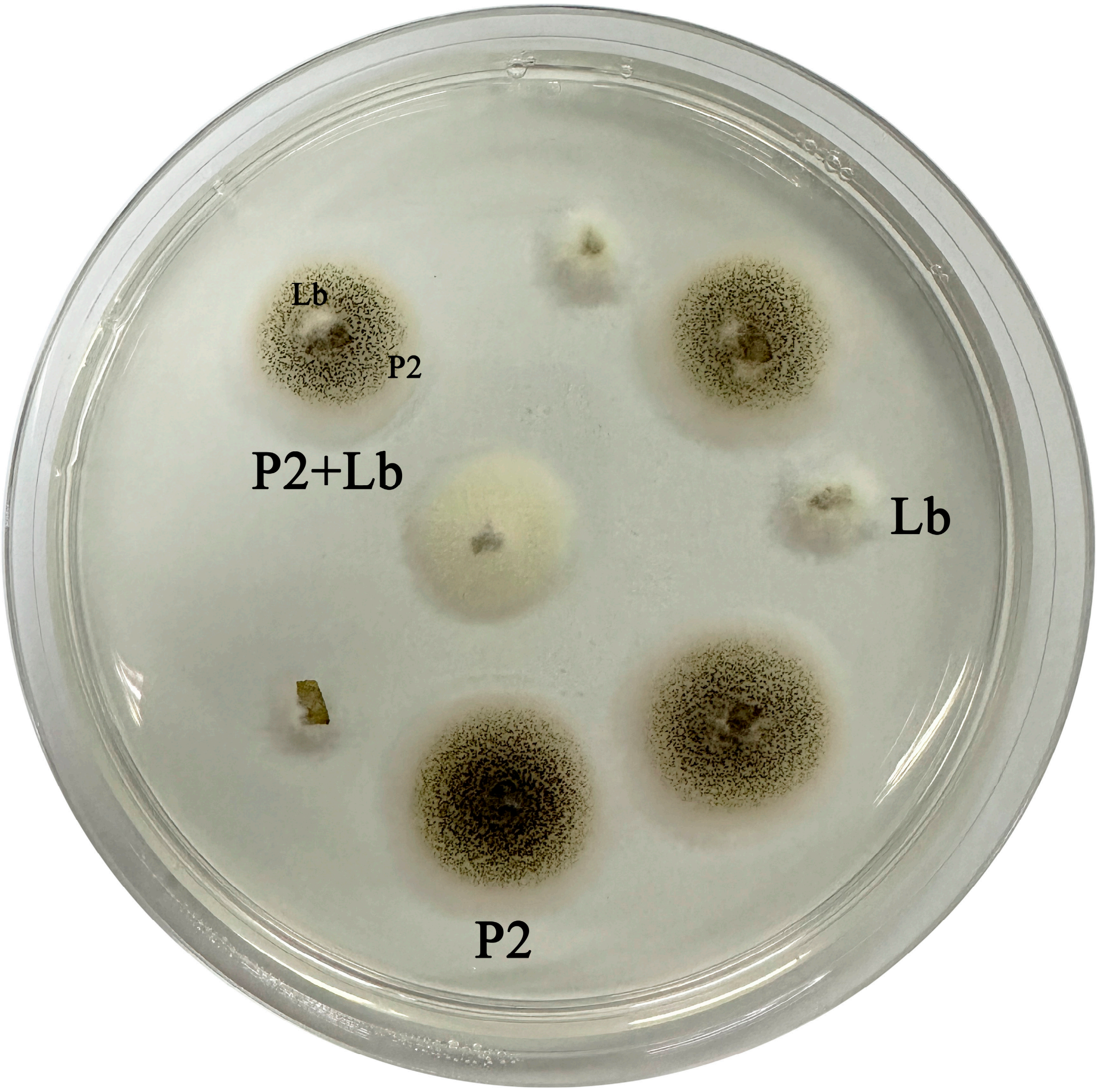

Figure S3. Isolation of *D. macrostoma* P2 and *L. biglobosa* Lb20 in rapeseed cotyledons. Note difference between the two fungi in morphology and size of the colonies

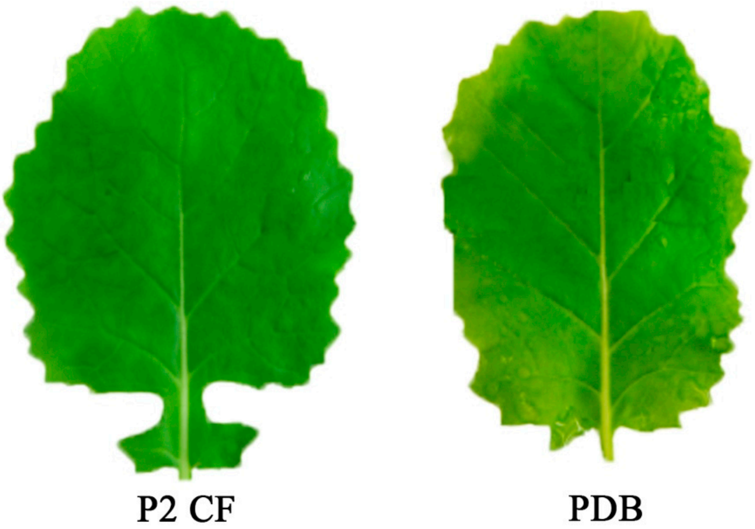

Figure S4. Two leaves of rapeseed treated with the culture filtrate (CF) of *D. macrostoma* P2 and PDB alone.

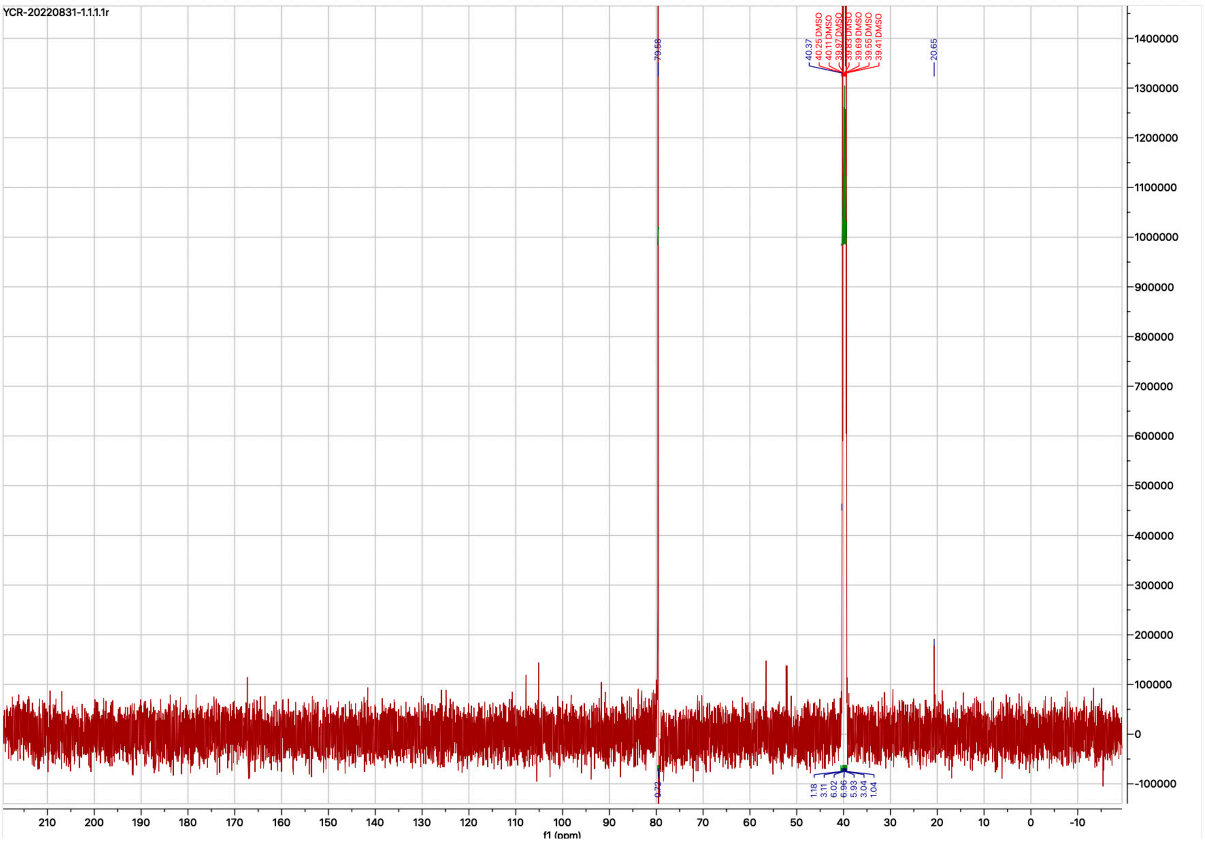

Figure S5. ^{13}C NMR (125 MHz, DSMOMeOH-d6, NMR) spectrum for penicillither from *Didymella macrostoma* P2.

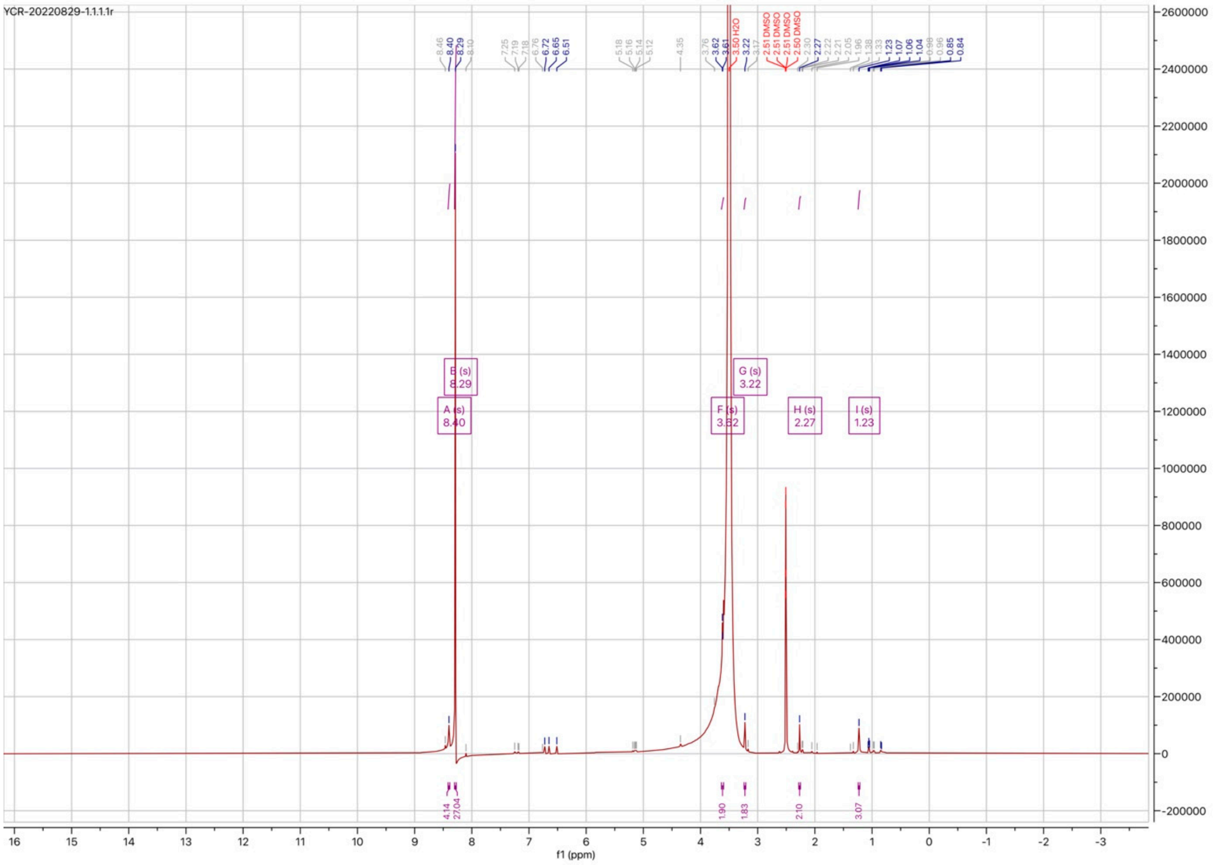

Figure S6. ^{1}H NMR (600 MHz, DSMO-d$_6$, NMR) spectrum for penicillither A from *Didymella macrostoma* P2.

Table S1. PCR primers used in this study for amplification of different genes.

Locus	Primer	Sequence (5' to 3')	Expected size	Reference
ITS-rDNA	V9G	TCCGTAGGTGAACCTGCGG	531 bp	(1)
	ITS4	TCCTCCGCTTATTGATATGC		(4)
28S rDNA (LSU)	LR0R	GTACCCGCTGAACTTAAGC	1319 bp	(3)
	LR7	TACTACCACCAAGATCT		
tub2	TUB2Fd	GTBCACCTYCARACCGGYCARTG	359 bp	(5)
	TUB4Rd	CCRGAYTGRCCRAARACRAAGTTGTC		
rpb2	fRPB2-5F	GAYGAYMGWGATCAYTTYGG	1132 bp	(2)
	fRPB2-7cR	CCCATRGCTTGYTTRCCCAT		

References: (1) de Hoog, G.S., and van den Ende, A.H.G. *Mycoses* 1998, 41: 183-189; (2) Liu, Y.J., *et al. Mol. Biol. Evol.* 1999, 16: 1799-1808; (3) Vilgalys, R., and Hester, M. *J. Bacteriol.* 1990, 172: 4238-4246; (4) White, T.J., *et al. In*: PCR Protocols: A Guide to Methods and Applications (Innis, M.A., *et al.* eds) Academic Press, San Diego, CA, USA, 1990, pp. 315-322; (5) Woudenberg, J.H.C., *et al. Persoonia* 2009, 22: 56-62.

Table S2. PCR thermal programs for amplification of different fungal genes.

PCR stage	Temperature	Time	Duration
For amplification of ITS-rDNA (P1 to P8, Lb20)			
Pre-denaturation	94°C	5 min	
denaturation	94°C	30 s	32 cycles
Annealing	56°C	30 s	
Extension	72°C	1 min	
Extension	72°C	10 min	
drop temperature	16°C	5 min	
For amplification of LSU-rDNA (P1 to P8, Lb20)			
Pre-denaturation	94°C	5 min	
denaturation	94°C	30 s	33 cycles
Annealing	50°C	1 min	
Extension	72°C	2 min	
Extension	72°C	10 min	
drop temperature	16°C	5 min	
For amplification of *tub2* (P1 to P8, Lb20)			
Pre-denaturation	95°C	5 min	
denaturation	95°C	30 s	35 cycles
Annealing	52°C	30 s	
Extension	72°C	30 s	
Final extension	72°C	10 min	
drop temperature	16°C	5 min	
For amplification of *rpb2* (P1 to P8, Lb20)			
Pre-denaturation	94°C	5 min	
denaturation	94°C	45 s	30 cycles
Annealing	54°C	45 s	
Extension	72°C	2 min	
Final extension	72°C	8 min	
drop temperature	16°C	5 min	

Table S3. Origin of the fungal isolates used in this study and GenBank accession numbers.

Fungus and strain (old name) [a]	Host plant	Country	GenBank accession number [b]			
			ITS	LSU	*TUB2*	*RPB2*
Boeremia exigua var. *exigua* CBS 431.74 (*Ph. exigua* var. *exigua*)	*Solanum tuberosum*	The Netherlands	FJ427001	EU754183	FJ427112	KT389569
Coniohyrium paimarum CBS 400.71	*Chamaerops humilis*	Italy	AY720708	EU754153	KT389792	KT389592
Didymella bellidis CBS 714.85 (*Ph. bellidis*)	*Bellis perennis*	The Netherlands	GU237904	GU238046	GU237586	KP330417
Didymella glomerata CBS 528.66 (*Ph. glomerata*)	*Chrysanthemum* sp.	The Netherlands	FJ427013	EU754184	FJ427124	GU371781
Didymella macrostoma CBS 482.95 (*Ph. macrostoma*)	*Larix decidua*	Germany	GU237869	GU238099	GU237626	KT389609
Didymella macrostoma CBS 223.69 (*Ph. macrostoma*)	*Acer pseudoplatanus*	Switzerland	GU237801	GU238096	GU237623	KT389608
Didymella macropodii CBS 100190 (*Ph. nigrificans*)	*Brassica napus*	Germany	GU237708	GU237967	GU237530	N.D.
Didymella macropodii PD 84/512 (*Ph. nigrificans*)	A cruciferous plant	Unknown	GU237919	GU237966	GU237529	N.D.
Didymella molleriana CBS 229.79 (*Ph. digitalis*)	*Digitalis purpurea*	New Zealand	GU237802	GU238067	GU237605	KP330418
Phoma herbarum CBS 502.91 (*Phoma herbarum*)	*Nerium* sp.	Netherlands	GU237874	GU238082	GU237613	KP330419
Phoma herbarum CBS 615.75 (*Ph. herbarum*)	*Rose multiflora* cv. *cathayensis*	Netherlands	FJ427022	EU754186	FJ427133	KP330420
Leptosphaeria biglobosa CBS 532.66 (*Pl. biglobosa*)	*Brassica* sp.	Netherlands	KT389541	KT389759	KT389840	KT389668
Leptosphaeria biglobosa CBS 119951 (*Pl. biglobosus*)	*Brassica rapa*	Netherlands	JF740198	JF740274	N.D.	N.D.
Leptosphaeria biglobosa CBS 127249 (*Pl. biglobosus*)	*Brassica juncea*	France	JF740199	JF740275	N.D.	N.D.
Leptosphaeria maculans CBS 275.63 (*Pl. lingam*)	*Brassica* sp.	UK	JF740234	JF740306	KT389841	KT389669

[a]CBS: Centraalbureau voor Schimmelcultures (Utrecht, The Netherlands); IBCN, International Blackleg of Crucifers Network (U.S.A); PD, Plant Protection Service (Wageningen, The Netherlands);

[b]ITS, internal transcribed spacer regions 1 and 2 including 5.8S nrDNA gene; LSU, 28S large subunit of the nrRNA gene; *rpb2*, RNA polymerase II second largest subunit; *tub2*: β-tubulin. N.D., Not Determined.
